# Chiral Diketopyrrolopyrrole-Helicene Polymer With Efficient Red Circularly Polarized Luminescence

**DOI:** 10.3389/fchem.2020.00237

**Published:** 2020-04-09

**Authors:** Kais Dhbaibi, Chengshuo Shen, Marion Jean, Nicolas Vanthuyne, Thierry Roisnel, Marcin Górecki, Bassem Jamoussi, Ludovic Favereau, Jeanne Crassous

**Affiliations:** ^1^Univ Rennes, CNRS, Institut des Sciences Chimiques de Rennes, ISCR-UMR 6226, Rennes, France; ^2^Faculty of Science of Gabès, University of Gabès, Gabes, Tunisia; ^3^State Key Lab of Metal Matrix Composites, School of Chemistry and Chemical Engineering, Shanghai Jiao Tong University, Shanghai, China; ^4^Aix Marseille University, CNRS, Centrale Marseille, iSm2, Marseille, France; ^5^Dipartimento di Chimica e Chimica Industriale, University of Pisa, Pisa, Italy; ^6^Institute of Organic Chemistry, Polish Academy of Sciences, Warsaw, Poland; ^7^Department of Environmental Sciences, Faculty of Meteorology, Environment and Arid Land Agriculture, King Abdulaziz University, Jeddah, Saudi Arabia

**Keywords:** helicene, diketopyrrolopyrrole, circularly polarized luminescence, red emitters, exciton coupling, chiral polymers

## Abstract

Chiral diketopyrrolopyrrole (DPP)-helicene polymers were synthesized to develop efficient red circularly polarized (CP) light emitters. These original chiral dyes display intense electronic circular dichroism (ECD) and CP luminescence (CPL) in the far-red spectral region owing to the presence of excitonic coupling between achiral DPPs within the chiral environment of the polymeric structure. This work affords an interesting example illustrating the potential of π-conjugated helical polymers for chiral optoelectronic applications.

## Introduction

**Figure 1:**
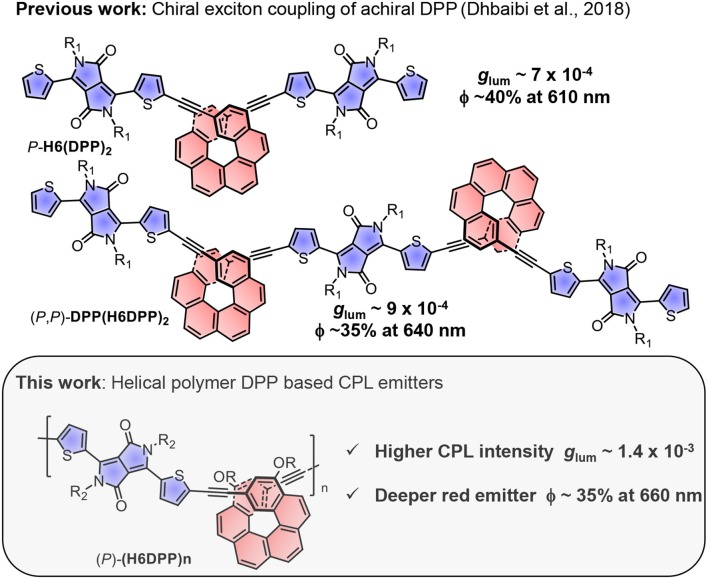
Chemical structures of CPL emitters based on helicene-diketopyrrolopyrrole polymers and their corresponding polarized and unpolarized luminescence characteristics (*P* enantiomers are shown).

Circularly polarized (CP) light has received renewed attention owing to its superior potential over unpolarized one in a diverse range of domains such as (chir)optoelectronics (stereoscopic displays, organic light-emitting diodes (OLEDs), optical information processing, etc.) as well as in bio-imaging and chiral sensing (Riehl and Richardson, 1986; Berova et al., 2000, 2012; Carr et al., 2012; Maeda and Bando, 2013; de Bettencourt-Dias, 2014; Kumar et al., 2015a; Zinna and Di Bari, 2015, 2018; Zinna et al., 2015, 2017; Brandt et al., 2016; Longhi et al., 2016; Li et al., 2017; Han et al., 2018; Tanaka et al., 2018). Until recently, luminescent chiral lanthanides complexes have been the most studied molecular CPL emitters since this family of compounds can display relatively high level of circularly polarized emission, characterized by a luminescence dissymmetry factor *g*_lum_ = 2(*I*_L_-*I*_R_)/(*I*_L_+*I*_R_), of more than 1 (Carr et al., 2012; Zinna et al., 2015; Zinna and Di Bari, 2018). However, lanthanide complexes often possess low luminescent quantum yield (ϕ) and stability issues, which may difficultly render their integration in optoelectronic devices such as CP-OLEDs, chiral photovoltaics and transistors for example. To circumvent these aspects, the development of chiral emitting small organic molecules (SOM) has gained increasing interest, also benefiting from their tunable photophysical and chiroptical properties from the blue to the near-infrared spectral region (Li et al., 2017; Han et al., 2018). One particularly appealing synthetic strategy to design efficient CPL emitters has consisted in developing chirally perturbed π-extended achiral chromophores, mostly based on *C*_2_-symmetric chiral moieties (chiral binaphthyl or 1,2-diamino-cyclohexane derivatives) linked to bodipy or perylene organic dyes (Tsumatori et al., 2010; Langhals et al., 2011; Kumar et al., 2013, 2014, 2015b; Sánchez-Carnerero et al., 2014; Sheng et al., 2016). In addition, helicenes have recently shown to be very good scaffolds for the development of emissive materials with strong CPL activity (Gingras, 2013; Chen and Shen, 2016; Dhbaibi et al., 2018, 2020; Zhao et al., 2019; Shen et al., 2020). Following this approach, we recently reported helical π-conjugated helicene-diketopyrrolopyrrole (DPP) dyes [*P*-**H6(DPP)**_**2**_ and (*P*,*P*)-**DPP(H6DPP)**_**2**_, Figure 1] (Dhbaibi et al., 2018) as red CPL emitters, (Shen et al., 2014; Saleh et al., 2015; Pascal et al., 2016; Sakai et al., 2016; Biet et al., 2017; Nishimura et al., 2017) arising from an intramolecular exciton coupling (Berova et al., 2012) between the achiral DPP units placed within the chiral environment of the helicene. Based on purely π-π^*^ transitions, this design afforded promising *g*_lum_ factors of 6–9 × 10^−4^ at 610–650 nm associated with fluorescence quantum yields (ϕ) of 35–40%. To our knowledge, this study remains the only example of DPP based CPL emitters, despite the promising potential of diketopyrrolopyrrole and its derivatives in a broad range of applications such as OLEDs, photovoltaic devices, organic transistors, and fluorescent probes (Nielsen et al., 2013; Grzybowski and Gryko, 2015; Heyer et al., 2015; Kaur and Choi, 2015; Data et al., 2016).

With the aim of deeper exploring this innovative and promising synergy between chiral helicene and achiral diketopyrrolopyrrole dye, we report here the synthesis and chiroptical properties of novel π-conjugated helical polymeric CPL emitters, namely *rac*-, (*P*)- and (*M*)-**(H6DPP)**_**n**_, Figure 1. These new examples display intense electronic circular dichroism (ECD) in the visible region and strong red CPL with *g*_lum_ = 1.4 × 10^−3^ at 660 nm, associated with a high ϕ of ~35 %. This first example of chiral helicene-DPP based polymer exhibits higher CPL response than the molecular chiral helicene-DPP dyes previously reported, and brings interesting aspects for the design of efficient polymeric red and near infra-red CPL emitters.

## Results and Discussion

### Synthesis of Polymer (H6DPP)_n_

**Figure 2:**
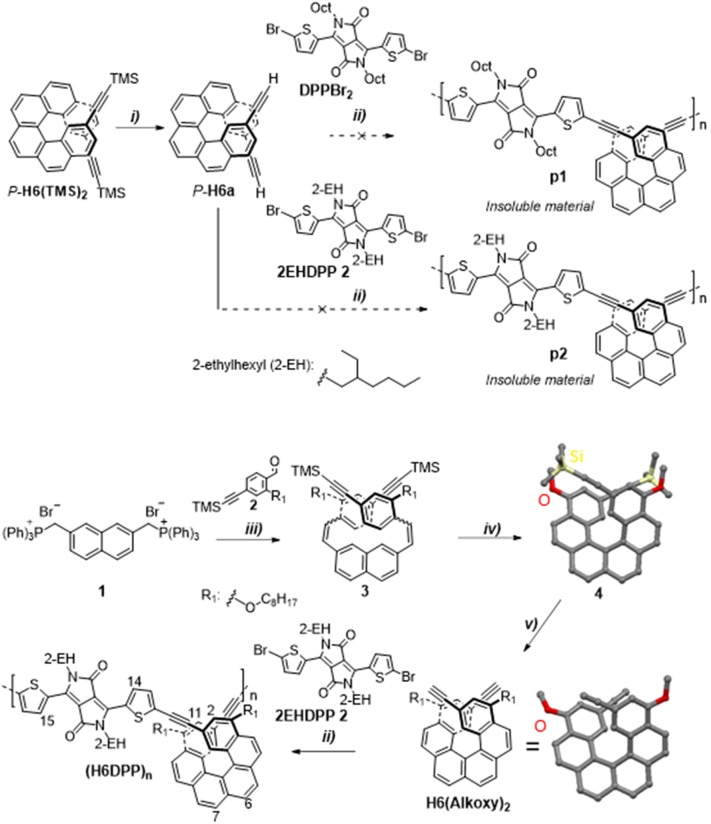
Synthesis of enantiopure *P*-**(H6DPP)**_**n**_. TMS: trimethylsilyl. Reaction conditions: (i) TBAF, CHCl_3_; (ii) Pd(PPh_3_)_4_, CuI, Et_3_N/toluene, 50°C; (iii) *n*-BuLi, THF,−78°C to rt, 60%; (iv) hν, *I*_2_ (1 equiv.), propylene oxide (50 equiv.), toluene, 50%; (v) TBAF, CHCl_3_, 52%, chiral HPLC. X-ray crystal structures of *rac*-**4** and *rac*-**H6(Alkoxy)**_**2**_ (octyl chains and hydrogen atoms have been omitted for clarity).

The helicene-DPP polymers were prepared using the Sonogashira coupling between a helicene decorated with two alkynyl functions and a DPP core substituted with two bromothiophene units. In a first attempt to synthesize polymer **(H6DPP)**_**n**_, the coupling was performed using enantiopure *P*- and *M*-2,15-bis-(ethynyl)[6]helicene (*P*- and *M*-**H6a**) (Anger et al., 2012) with 3,6-bis(5-bromothiophen-2-yl)-2,5-diketopyrrolopyrrole, **DPPBr**_**2**_, respectively (Figure 2, where only *P* enantiomer is described) (Wu et al., 2015). While the reactions seemed to proceed efficiently, they resulted in the formation of insoluble dark blue material (**p1**, Figure 2). To circumvent this solubility issue, we introduced widely used branched 2-ethylhexyl (2-EH) chains on the DPP unit in place of the linear octyl ones (Huo et al., 2009; Palai et al., 2010). Although this fragment has a chiral center, we used its racemic form since a weak influence of these additional stereogenic centers is expected on the photophysical and chiroptical properties of the final polymer in diluted solution. Unfortunately, this new approach also afforded insoluble blue solid when a stoichiometric mixture of *P*-**H6a** and **2EHDPP 2** was subjected to the Sonogashira coupling conditions (**p2**, Figure 2). To further increase the solubility of the obtained polymer material, the helicenic fragment was also functionalized with additional linear octyloxy chains through a new synthetic pathway involving 2-(octyloxy)-4-((trimethylsilyl)ethynyl)benzaldehyde, **2**, as starting material for the synthesis of the helicene fragment. Indeed, the latter was engaged in a double Wittig reaction with naphthyl-2,7-dimethylphosphonium bromide salt, **1**, to give 2,7-bis(2-(octyloxy)-4-((trimethylsilyl)ethynyl)styryl)naphthalene, **3**, in 60% yield. The resulting *cis*/*trans* stilbene mixture was subsequently submitted to a photocyclisation reaction with propylene oxide as an acid scavenger to afford *rac*-2,15-bis-((trimethylsilyl)ethynyl)-4,13-bis-(octyloxy)[6]helicene (**4**) in 50% yield, followed by deprotection reaction of the two remaining TMS groups to yield **H6(Alkoxy)**_**2**_ (**Scheme 1**). Fully characterized by NMR spectroscopy and mass spectrometry (see Supplementary Information), the structures of the two latters were further ascertained by X-ray crystallographic analysis (see **Scheme 1** and Supplementary Information). Both *rac*-**4** and *rac*- **H6(Alkoxy)**_**2**_ crystallized in a *P*-1 space group and displayed helicity (dihedral angle between the two terminal rings) of 41.1° and 54.4°, respectively, which is in the range of classical carbo[6]helicenes (Gingras, 2013; Chen and Shen, 2016; Dhbaibi et al., 2019). Moreover, the lateral octyloxy chains point toward the outside of the molecules, which may disfavor the formation of polymer aggregates in solution.

*Rac*-**H6(Alkoxy)**_**2**_ was then submitted to chiral HPLC separation to give *P*-(+) and *M*-(–) in 99 and 98.5% of *ee*, respectively (see Supplementary Information for detailed experimental conditions). These enantiomers, as well as the racemic compound, were finally engaged in the polymerization reaction with **2EHDPP 2** and gave expected soluble polymers which were firstly filtered over a silica plug, then further purified by size-exclusion chromatography (SEC, CHCl_3_) before precipitated using CHCl_3_/MeOH solvent mixture to yield *P*-, *M*-, and *rac*-**(H6DPP)**_n_ in ~45% yield for each polymer (see Supplementary Information). These novel chiral dyes are soluble in common organic solvents such as THF, CHCl_3_, and CH_2_Cl_2_, and were characterized by ^1^H NMR and SEC using a polystyrene standard in THF. The obtained NMR spectrum displays characteristic signals for both helicene and DPP starting materials: for instance, shielded H^2, 11^ and deshielded H^6, 7^ helicenic protons at 6.75 and 8.50 ppm respectively, and deshielded DPP protons H^14, 15^ at 9.10 ppm (see Supplementary Information). The number average molecular mass (*M*_n_) for *rac*-, *P*-, and *M*-**(H6DPP)**_**n**_ were estimated to be 6.9 ×10^3^, 5.3 ×10^3^, and 6.4 × 10^3^, respectively, which correspond to a low degree of polymerization, *ca*. 5-6 (helicene-DPP) units (see Supplementary Information). The thermal stability of *rac*-**(H6DPP)**_**n**_ was also evaluated by thermogravimetric analysis (TGA) and resulted in an onset decomposition temperature at 300°C with a 10% weight loss.

### UV-Vis, ECD, and Electrochemical Characterizations

The ground state photophysical and chiroptical properties of DPP-helicene polymers were investigated in CH_2_Cl_2_ solutions and compared with corresponding precursors and previously reported **DPP(H6DPP)**_**2**_. UV-Vis spectrum of **(H6DPP)**_**n**_ polymer displays two main absorption signatures between 300 and 425 nm and between 530 and 675 nm that correspond to a combination of helicene and DPP transitions for the higher energy region and only from DPP transitions for the low energy part. The latter absorption region is red-shifted by 70 nm in comparison with DPP precursor, characterized by ε = 7.0 × 10^4^ M^−1^ cm^−1^ at 565 nm and 35 × 10^4^ M^−1^ cm^−1^ at 635 nm for **2EHDPP 2** and **(H6DPP)**_**n**_, respectively, resulting from the extension of the π-conjugation via the alkynyl bridges between the DPP dye and the helicene units. These observations are supported by comparison with **DPP(H6DPP)**_**2**_ UV-vis spectrum, where contributions of both “DPP-helicene” and “helicene-DPP-helicene” fragments are superimposed in the red region (see Supplementary Information for additional details). Going from oligomer **DPP(H6DPP)**_**2**_ to polymer **(H6DPP)**_**n**_ does not strongly red-shift the overall absorption signature (λ_max_ = 620 and 635 nm for **DPP(H6DPP)**_**2**_ and **(H6DPP)**_**n**_ lowest absorption bands, respectively), which suggests that electronic communication between each bis(ethynyl)DPP unit through the π-conjugated helicene is relatively limited along the polymer. The observed difference of 15 nm results probably from the presence of the electron donating octyloxy groups on the helicene fragment for **(H6DPP)**_**n**_.

**Figure 3:**
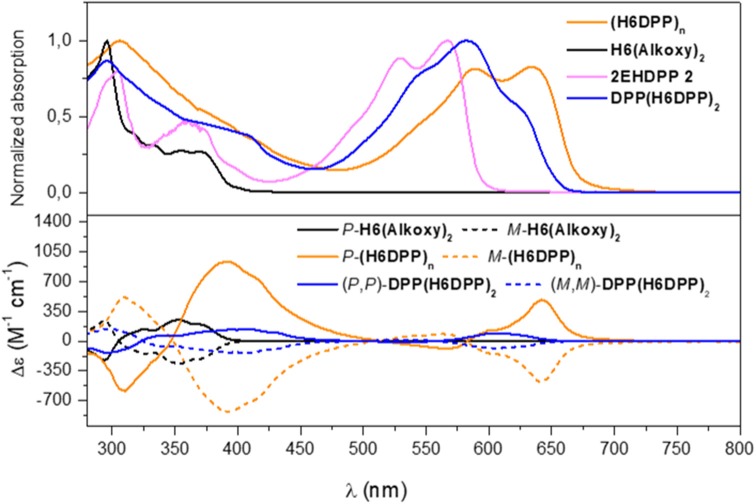
UV-vis (top) and ECD (bottom) spectra of **H6(Alkoxy)**_**2**_ (black), **2EHDPP 2** (purple), **DPP(H6DPP)**_**2**_ (blue) and **(H6DPP)**_**n**_ (orange) in CH_2_Cl_2_ at 298 K (~10^−5^ M).

**Figure 4:**
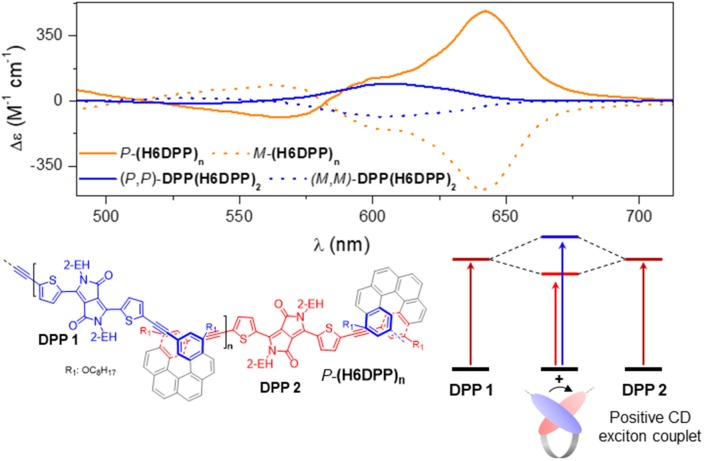
Enlargement of 490–710 nm region of the ECD spectra of **DPP(H6DPP)**_**2**_ (blue) and **(H6DPP)**_**n**_ (orange) in CH_2_Cl_2_ at 298 K (~10^−5^ M), with schematic illustration of the chiral exciton coupling process in **(H6DPP)**_**n**_.

ECD of *P*- and *M*-**(H6DPP)**_**n**_ displays expected mirror-image spectra with intense responses ranging from 280 to 700 nm (Figure 3). *P*-**(H6DPP)**_**n**_ exhibits an intense negative ECD band (Δε = −576 M^−1^ cm^−1^) at 309 nm which is 19 nm red-shifted compared to helicene *P*-**H6(Alkoxy)**_**2**_, a broad strong positive band between 347 and 500 nm with a maximum at 391 nm (Δε = + 940 M^−1^ cm^−1^) and a shoulder at 414 nm (Δε = + 740 M^−1^ cm^−1^), a negative contribution at 565 nm (Δε = −88 M^−1^ cm^−1^) followed by a positive one with a maximum at 640 nm (Δε = + 481 M^−1^ cm^−1^). This lowest bisignate DPP-centered signal is clearly reminiscent of what we observed for *(P,P)*-**DPP(H6DPP)**_**2**_ (Figure 4), which was attributed to a chiral excitonic coupling between the DPP-centered π-orbitals in the helical arrangement (Bouvier et al., 2018; Dhbaibi et al., 2018, 2020). Elongating the number of helicene-DPP association within polymer *P*-**(H6DPP)**_**n**_ appears as an efficient strategy to obtain very intense ECD signature across the whole spectrum and especially in the red region thanks to the increase of the excitonic coupling intensity. Indeed, this higher sensitivity to red circularly polarized light for *P*-**(H6DPP)**_**n**_ is confirmed by the evaluation of the associated dissymmetry factor *g*_abs_ = Δε/ε = +1.8 × 10^−3^ at 649 nm, i.e., a 40 % increase in comparison with (*P,P*)-**DPP(H6DPP)**_**2**_ (*g*_abs_ = +1.3 × 10^−3^ at 610 nm).

The electrochemical behavior of **(H6DPP)**_**n**_ was investigated by cyclic voltammetry (CV, Figure S17 and Table S1), which displays pseudo-reversible oxidation process at ca. +0.81 V and a reversible reduction one at −1.24 V vs. SCE, respectively assigned to the oxidation and the reduction of the DPP unit(s). The calculated HOMO and LUMO energy level were −5.21 eV and a LUMO level of −3.16 eV, respectively, leading to an estimated electrochemical band gap of 2.05 eV.

### Unpolarized (PL) and Circularly Polarized Luminescence (CPL)

To our delight, **(H6DPP)**_**n**_ displays intense unpolarized vibronic emission arising from the DPP-ethynyl fragment with a maximum intensity at 660 nm and a quantum yield of 35%. Interestingly, these values suggest that embedding DPP-helicene fragment within a polymer material is also an efficient strategy to make deeper red emitter while keeping a high fluorescence efficiency since **DPP(H6DPP)**_**2**_ exhibits similar luminescence quantum yield (ϕ = 35%) but its emission is blue shifted (λ_max_ = 650 nm). In order to rule out the possibility of charge transfer character the emission of the polymer was carried out in solvents of different polarity including toluene, tetrahydrofuran, and dichloromethane in which the polymer was fully soluble (Figure S13). A similar spectral behavior was found in these solvents with a structured signals and no significant shift of the emission spectra, indicating that the nature of the emission is mainly based on π-π^*^ transitions localized on the DPP units. The fluorescence kinetics of the polymer was also performed at 650 nm (Figure S14) and fits a single-exponential decay function with a decay time constant of 2.08 ns.

**Figure 5:**
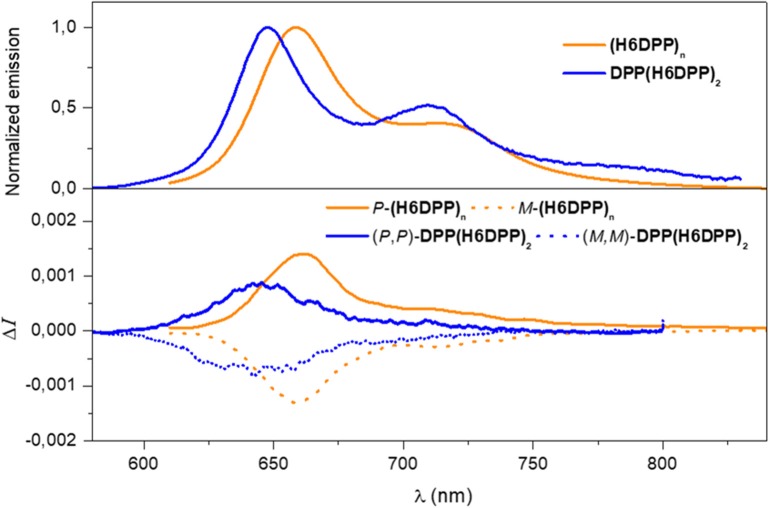
Normalized fluorescence (top) and CPL (bottom) spectra of **DPP(H6DPP)**_**2**_ (blue) and **(H6DPP)**_**n**_ (orange) in CH_2_Cl_2_ at 298 K. CPL spectra for *P* and *M* enantiomers are shown, respectively, in solid and dotted lines.

Regarding circularly polarized luminescence (CPL), mirror-image spectra were also obtained for *P*- and *M*-**(H6DPP)**_**n**_ with a maximum and a structural signature similar to unpolarized emission, highlighting the synergy of the DPP-helicene association also in the polymer chiral excited-state (Figure 5). Moreover, *g*_lum_ factor of +1.3 × 10^−3^ was determined for *P*-**(H6DPP)**_**n**_, which suggests a relatively similar chiral geometry of the ground and emitting excited states (*g*_lum_/*g*_abs_= 0.72) and represents also a *ca*. 40% increase in comparison with *(P,P)*-**DPP(H6DPP)**_**2**_ (*g*_lum_ = + 0.9 × 10^−3^). The obtained *g*_lum_ values for polymers **(H6DPP)**_**n**_ are in the same range of reported SOM CPL emitters (10^−4^-10^−2^), and are among the most efficient ones for the far red and near infrared region (i.e., for λ_max_ > 650 nm, 3 × 10^−4^ < *g*_lum_ < 4.3 × 10^−3^) (Tanaka et al., 2018).

## Conclusions

In summary, we have successfully prepared novel helical conjugated polymers based on the association between enantiopure carbo[6]helicene derivative and diketopyrrolopyrrole dye via the Sonogashira cross-coupling. These polymers show strong ECD signal in the visible region (Δε × 482 M^−1^ cm^−1^ at 642 nm, for *P*-**(H6DPP)**_**n**_) and strong CPL emission response signals (*g*_lum_ = +1.3 × 10^−3^ at 662 nm) along with high fluorescence quantum efficiency (φ_f_ = 35 %). Extending the efficiency of exciton coupling process in chiral polymers allow efficient preparation of CPL emitters deeper in the red and near-infrared region. Our results provide an alternative approach to the metalation and the push-pull functionalization methodologies to extend and to improve the chiroptical responses of the helicene molecules by taking advantages of the strong synergy between the chiral environment controlled by the helicene center and the interesting photophysical properties offered by the corresponding dye. The polymers based on the helicene unit that we presented in this work can be used as a new class of candidates for efficient CPL materials in optoelectronics and bioimaging applications.

## Data Availability Statement

All datasets generated for this study are included in the article/Supplementary Material.

## Author Contributions

KD and CS synthesized and characterized the molecules. MJ and NV performed the HPLC resolution. TR performed the X-ray analysis. MG performed CPL measurements. BJ, LF, and JC supervised the work, analyzed the results and wrote the manuscript.

### Conflict of Interest

The authors declare that the research was conducted in the absence of any commercial or financial relationships that could be construed as a potential conflict of interest.
