# Supplementary material for: Chiral Diketopyrrolopyrrole-Helicene Polymer With Efficient Red Circularly Polarized Luminescence
Source: Front Chem. 2020 Apr 9;8:237. doi: 10.3389/fchem.2020.00237 (PMC7160364; doi:10.3389/fchem.2020.00237)
Supplement: Supplementary file 1 [file Data_Sheet_1.PDF]

# Electronic Supporting Information

# Chiral diketopyrrolopyrrole-helicene polymer with efficient red circularly polarized luminescence

Kais Dhbaibi,[a,b] Chengshuo Shen,[a,f] Marion Jean,[c] Nicolas Vanthuyne,[c] Thierry Roisnel,[a] Marcin Górecki,[d,g] Bassem Jamoussi,[e] Ludovic Favereau,[a,]* and Jeanne Crassous[a,]*

[a] Univ Rennes, CNRS, Institut des Sciences Chimiques de Rennes, ISCR-UMR 6226, F-35000 Rennes, France.

[b] University of Gabès, Faculty of Science of Gabès, Zrig, 6072 Gabès, Tunisia.

[c] Aix Marseille University, CNRS, Centrale Marseille, iSm2, Marseille, France.

[d] Dipartimento di Chimica e Chimica Industriale, University of Pisa, Pisa, Italy.

[e] Department of Environmental Sciences,Faculty of Meteorology, Environment and Arid Land Agriculture, King Abdulaziz university, Jeddah, Saudi Arabia.

[f] School of Chemistry and Chemical Engineering, State Key Lab of Metal Matrix Composites, Shanghai Jiao Tong University, Shanghai 200240, China

[g] Institute of Organic Chemistry, Polish Academy of Sciences, Warsaw, Poland.

## Table of Contents

A. General method………………………………………………S2
B. Synthetic procedures……………………………………………S3
C. NMR spectra………………………………………………..……..S8
D. GPC data……………………………………………………......S16
E. Photophysical characterization…………….…………..…S17
F. Electrochemical Measurement……………………..…..….S18
G. Thermal properties……………………………….…..…S19
H. HPLC separations…….………………………………….S21
I. X-ray crystallographic data……………………………..S23
J. References…………………..…………………………....S25

# A. General method

$^{1}H$ and $^{13}C$ NMR spectra were recorded at room temperature on an *AVANCE III 400 BRUKER* or an *AVANCE I 500 BRUKER* at Centre Régional de Mesures Physiques de l'Ouest (CRMPO), Université de Rennes 1. Chemical shifts δ are given in ppm and coupling constants *J* in Hz. Chemical shifts for $^{1}H$ NMR spectra are referenced relative to residual protium in the deuterated solvent (δ = 7.26 ppm, $CDCl_3$). $^{13}C$ shifts are referenced to $CDCl_3$ peaks at δ = 77.16 ppm.

High-resolution mass (HR-MS) determinations were performed at CRMPO on a Bruker MaXis 4G by ASAP (+ or -) or ESI with $CH_2Cl_2$ as solvent techniques. Experimental and calculated masses are given with consideration of the mass of the electron.

UV-Visible (UV-vis, in $M^{-1}$ $cm^{-1}$) absorption spectra were recorded on a UV-2401PC Shimadzu spectrophotometer. Fluorescence spectra were recorded on a FL 920 Edinburgh fluorimeter.

Fluorescence quantum yields Φ were measured in diluted solution using the following equation:

$$\frac{\Phi_x}{\Phi_r} = \left(\frac{\mathrm{A}_r(\lambda)}{\mathrm{A}_x(\lambda)}\right)\left(\frac{n_x^2}{n_r^2}\right)\left(\frac{\mathrm{D}_x}{D_r}\right)$$

where: A(λ) is the absorbance at the excitation wavelength λ, *n* is the refractive index, D is the integrated intensity, and "r" and "x" stand for reference and sample, respectively. The fluorescence quantum yields were measured relative to rhodamine 6G in ethanol (Φ= 0.91).[1] Excitation of reference and sample compounds was performed at the same wavelength.

Electrochemical measurements were performed with a potentiostat-galvanostat AutoLab PGSTAT 302N controlled by resident GPES (General Purpose Electrochemical System 4.9) software using a conventional single-compartment three-electrode cell. The working and auxiliary electrodes were platinum electrodes and the reference electrode was the saturated potassium chloride calomel electrode (SCE). The supporting electrolyte was 0.1 N $Bu_4NPF_6$ (tetrabutylammonium hexafluorophosphate) in dichloromethane and solutions were purged with argon before the measurements. All potentials are quoted relative to SCE. In the experiments, the scan rate was either 100 or 200 mV/s

Electronic circular dichroism (ECD, in $M^{-1}$ $cm^{-1}$) was measured on a Jasco J-815 Circular Dichroism Spectrometer (IFR140 facility - Biosit - Université de Rennes 1).

The circularly polarized luminescence (CPL) measurements were performed using a home-built CPL spectrofluoropolarimeter (constructed with the help of the JASCO Company). The samples were excited using a 90° geometry with a Xenon ozone-free lamp 150 W LS. The following parameters were used: emission slit width ≈ 2 mm, integration time = 4 sec, scan speed = 50 nm/min, accumulations = 5. The concentration of all the samples was ca. $10^{-6}$ M. Excitation of the samples was performed at 350 nm.

Thin-layer chromatography (TLC) was performed on aluminum sheets precoated with Merck 5735 Kieselgel 60F254. Column chromatography was carried out with Merck 5735 Kieselgel 60F (0.040-0.063 mm mesh). Chemicals were purchased from Sigma-Aldrich, Alfa Aesar or TCI Europe and used as received.

Gel permeation chromatography **:** mass average molar mass (Mw), number average molar mass (Mn) and polydispersity index (PDI = Mw / Mn) values were determined by size-exclusion chromatography (SEC) in THF at 30 °C (flow rate = 1.0 mL min$^{-1}$) on a Polymer Laboratories PL50 apparatus equipped with a refractive index detector and a set of two ResiPore PLgel 3 µm MIXED-D 300 × 7.5 mm columns. The polymer samples were dissolved in THF (2 mg mL$^{-1}$). All elution curves were calibrated with polystyrene standards: Mn, SEC values were uncorrected for the possible difference in hydrodynamic radius vs. polystyrene.

Chemicals were purchased from Sigma-Aldrich, Alfa Aesar or TCI Europe, and used as received.

Compound 3,6-bis(5-bromothiophen-2-yl)-2,5-bis(2-ethylhexyl)pyrrolo[3,4-*c*]pyrrole-1,4(2*H*,5*H*)-dione **2EHDPP 2** was prepared using previously reported procedures.[2]

## B. Synthetic procedures

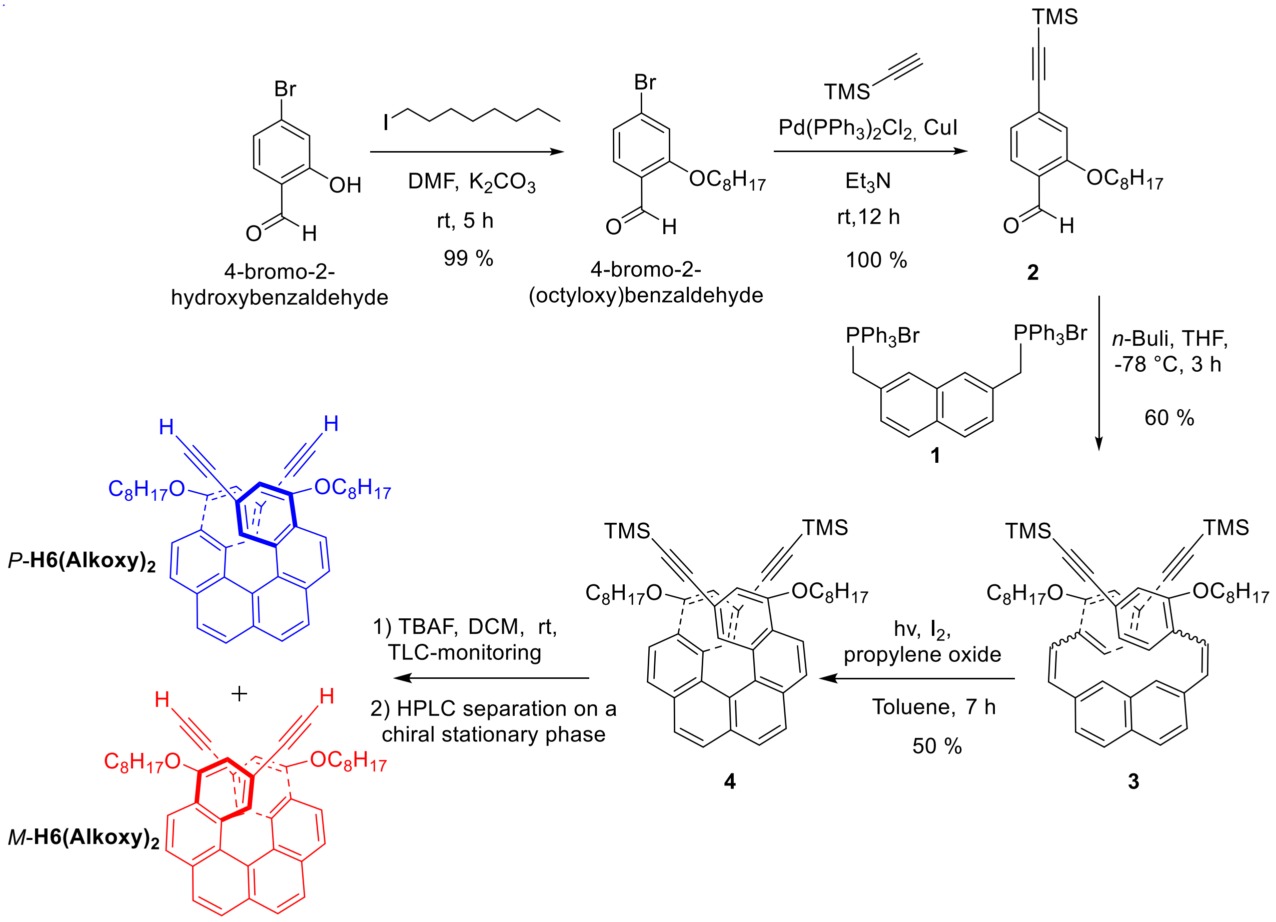


**Scheme S1.** Synthesis of **H6(Alkoxy)2** and separation of the corresponding enantiomers using chiral HPLC (see part H for details).

**4-Bromo-2-(octyloxy)benzaldehyde**

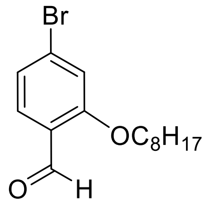


4-Bromo-2-hydroxybenzaldehyde (3.02 g, 15.0 mmol), 1-iodooctane (7.2 g, 30.0 mmol), and $K_2CO_3$ (4.14 g, 30.0 mmol) were suspended in dry DMF (50 mL). The reaction mixture was stirred for 5 h at room temperature, and then water (200 mL) was added into the reaction mixture and the organic phase extracted by $CH_2Cl_2$ (200 mL). The latter was concentrated under vacuum and the residue was purified by silica column chromatography (first 100 % heptane to remove the unreacted 1-iodooctane and then 100 % $CH_2Cl_2$) to afford the desired product as white solid (4.67 g, 99%).

**$^1$H NMR (400 MHz, Chloroform-*d*)** $\delta$ 10.43 (s, 1H), 7.68 (d, $J$ = 8.1 Hz, 1H), 7.15 (d, $J$ = 8.1 Hz, 1H), 7.14 (s, 1H), 4.06 (t, $J$ = 6.5 Hz, 2H), 1.85 (quint, $J$ = 6.5 Hz, 2H), 1.48 (quint, $J$ = 6.6 Hz, 2H), 1.41-1.22 (m, 8H), 0.89 (t, $J$ = 6.8 Hz, 3H).

**$^{13}$C NMR (101 MHz, Chloroform-*d*)** $\delta$ 189.0, 161.8, 130.6, 129.5, 124.1, 123.9, 116.3, 69.2, 31.9, 29.4, 29.3, 29.1, 26.1, 22.8, 14.2.

**2-(Octyloxy)-4-((trimethylsilyl)ethynyl)benzaldehyde (2)**

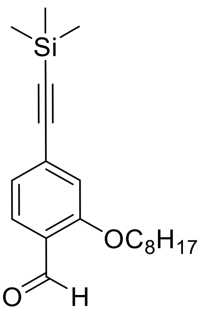


4-Bromo-2-octyloxybenzaldehyde (4.60 g, 14.7 mmol), $Pd(PPh_3)_2Cl_2$ (281 mg, 0.400 mmol), and CuI (76 mg, 0.400 mmol) were dissolved in triethylamine (20 mL) which was freed from oxygen by bubbling argon for 1 hour. Then, ethynyltrimethylsilane (4 mL) was added into the solution. The reaction was stirred at room temperature for 16 h under argon. After the reaction,

solvent was stripped off and the residue was purified by silica column chromatography ($CH_2Cl_2$) to afford the product as white solid (4.84 mg, 100%).

**$^1$H NMR (400 MHz, Chloroform-*d*)** $\delta$ 10.47 (d, J = 0.8 Hz, 1H), 7.77 (d, J = 7.9 Hz, 1H), 7.23 – 6.95 (m, 2H), 4.08 (t, J = 6.4 Hz, 2H), 1.94 – 1.79 (m, 2H), 1.56 – 1.23 (m, 10H), 0.90 (dt, J = 7.1, 3.3 Hz, 3H), 0.28 (s, 9H).

**$^{13}$C NMR (101 MHz, Chloroform-*d*)** $\delta$ 189.3, 161.2, 130.5, 128.2, 124.7, 124.3, 115.9, 104.2, 98.6, 68.9, 31.9, 29.4, 29.3, 29.2, 26.2, 22.8, 14.2.

**2,7-Bis(2-(octyloxy)-4-((trimethylsilyl)ethynyl)styryl)naphthalene (3)**

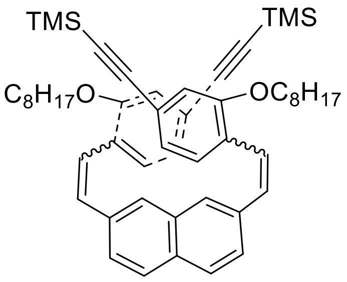


**3**

Naphthalene-2,7-diylbis(triphenylphosphonium)bromide **(1)** (500 mg, 0.6 mmol) was suspended in of dry THF (30 mL) under argon and cooled to −78 °C. *n*-Butyllithium (1.6 mol.L$^{-1}$ in hexane) (0.93 mL , 1.5 mmol, 2.5 equiv.) was added, and the reaction mixture was stirred for 5 min at −78 °C and then for 30 min at room temperature and the reaction turned to red. Then the reaction mixture was cooled to −78 °C again and 2-(octyloxy)-4-((trimethylsilyl)ethynyl)benzaldehyde **(2)** (434 mg, 1.2 mmol) dissolved in dry THF (10 mL) was added dropwise. The reaction was stirred at −78 °C for 5 min then at room temperature for 3 h. The solvent was stripped off in vacuum and the product was filtered over Celite and concentrated under reduced pressure. The residue was purified by column chromatography over silica gel with hexane/dichloromethane (9/1) to afford the stilbene derivatives as yellow solid (281 mg; 60%; *cis* and *trans*), which was used directly as an isomeric mixture for the next step.

**$^1$H NMR (400 MHz, Chloroform-d)** δ 7.87 – 7.77 (m, 4H), 7.77 – 7.69 (dd, J = 8.6, 1.6 Hz, 2H), 7.64 – 7.55 (m, 4H), 7.40 – 7.31 (d, *J* = 16.5 Hz, 2H), 7.15 – 7.09 (dd, *J* = 8.0, 1.4 Hz, 2H), 7.07 – 6.98 (d, *J* = 1.4 Hz, 2H), 4.13 – 4.03 (t, *J* = 6.5 Hz, 4H), 1.98 – 1.87 (m, 4H), 1.63 – 1.51 (m, *J* = 6.8 Hz, 4H), 1.50 – 1.24 (m, 16H), 0.95 – 0.86 (m, 6H), 0.34 – 0.26 (s, 18H).

**$^{13}$C NMR (101 MHz, Chloroform-d)** δ 156.1, 135.8, 134, 132.6, 130.5, 129.9, 128.1, 127.2, 126.8, 126.3, 124.6, 123.6, 123.5, 122.9, 115.3, 105.4, 94.8, 68.7, 31.9, 29.4, 29.3, 29.3, 26.2, 22.7, 14.1, 0.3.

**HR-MS** Bruker MaXis 4G, ASAP (+), 360 °C; ion $[M]^{+}$, $C_{52}H_{68}O_2Si_2$, m/z calculated 780.47579, m/z experimental 780.4757 (Δ=1 ppm).

***rac*-((9,16-Bis(octyloxy)hexahelicene-11,14-diyl)bis(ethyne-2,1-diyl))bis(trimethylsilane) (4)**

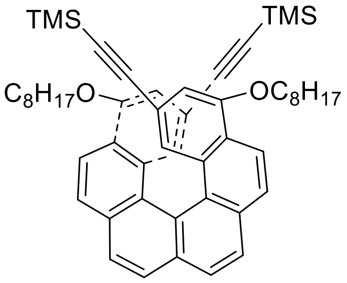


**4**

Stilbene derivative **(5)** (281 mg, 0.36 mmol) was dissolved in toluene (3 L) together with propylene oxide (1.2 mL, 18.00 mmol) and iodine (100 mg, 0.40 mmol. The solution was irradiated under mercury lamp for 7 h. The solvent was stripped off and the residue was purified by silica column chromatography heptane/dichloromethane (9/1) to afford the product **(4)** as yellow solid (145 mg, 50%).

**$^{1}$H NMR (400 MHz, Chloroform-d)** δ 8.46 – 8.39 (dd, *J* = 8.7, 0.9 Hz, 2H), 8.05 – 7.92 (m, 6H), 7.35 – 7.31 (t, *J* = 1.0 Hz, 2H), 6.71 – 6.68 (d, *J* = 1.3 Hz, 2H), 4.22 – 4.14 (dt, *J* = 9.0, 6.4 Hz, 2H), 4.10 – 4.02 (dt, *J* = 8.9, 6.7 Hz, 2H), 2.06 – 1.88 (m, 4H), 1.67 – 1.52 (m, 4H), 1.51 – 1.20 (m, 16H), 0.99 – 0.83 (m, 6H), 0.21 – 0.13 (s, 18H).

**$^{13}$C NMR (101 MHz, Chloroform-d)** δ 154.3, 133.1, 131.2, 130.3, 127.2, 127.1, 127.1, 126.2, 125.6, 124.5, 123.8, 121.4, 118.9, 107.2, 106.2, 92.4, 68.4, 31.8, 30.2, 29.5, 29.4, 29.2, 26.3, 22.7, 14.1.

**HR-MS** Bruker MaXis 4G, ASAP (+), 360 °C; ion $[M]^{+}$, $C_{52}H_{64}O_2Si_2$, m/z calculated 776.44449, m/z experimental 776.4447 (Δ=1 ppm).

***rac*-11,14-Diethynyl-9,16-bis(octyloxy)hexahelicene (H6(Alkoxy)$_2$)**

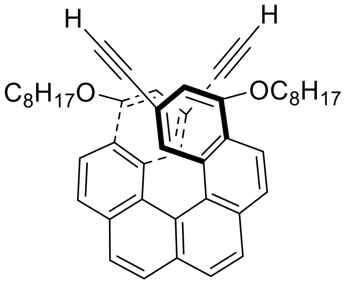


**H6(Alkoxy)$_2$**

Compound *rac*-**(4)** (50 mg ,0.064 mmol) and TBAF were solubilized in dichloromethane and stirred at room temperature the progress of the reaction was carefully monitored by TLC after addition of each 3 drops of TBAF solution. The reaction mixture was quenched by water (10 mL) and extracted by $CH_2Cl_2$ (10 mL). The organic layer was concentrated and purified by silica column chromatography ($CH_2Cl_2$) to afford **H6(Alkoxy)₂** as yellow solid (37 mg, 92%).

**$^1$H NMR (300 MHz, Chloroform-d)** δ 8.47 – 8.40 (d, *J* = 8.8 Hz, 2H), 8.05 – 7.93 (m, 6H), 7.33 – 7.28 (s, 2H), 6.80 – 6.69 (d, *J* = 1.3 Hz, 2H), 4.27 – 4.04 (m, 4H), 2.76 – 2.66 (s, 2H), 2.04 – 1.91 (p, *J* = 6.6 Hz, 4H), 1.68 – 1.21 (m, 20H), 1.01 – 0.82 (q, *J* = 10.8, 8.2 Hz, 6H).

**$^{13}$C NMR (101 MHz, Chloroform-d)** δ 154.5, 133.1, 131.8, 130.5, 127.3, 127.2, 127.0, 126.4, 125.1, 124.4, 124.1, 21.4, 117.9, 108.2, 84.2, 75.5, 68.7, 31.9, 31.45, 30.2, 29.7, 29.4, 29.3, 29.1, 26.3, 22.7, 14.2, 14.1.

**HR-MS** Bruker MaXis 4G, ASAP (+), 360 °C; ion $[M+Na]^+$, $C_{46}H_{48}O_2Na$, m/z calculated 655.35465, m/z experimental 655.3551 (Δ=1 ppm).

**General procedure of the polymerization reaction of Polymers (H6DPP)ₙ (*P*, *M* and *Racemic*):**

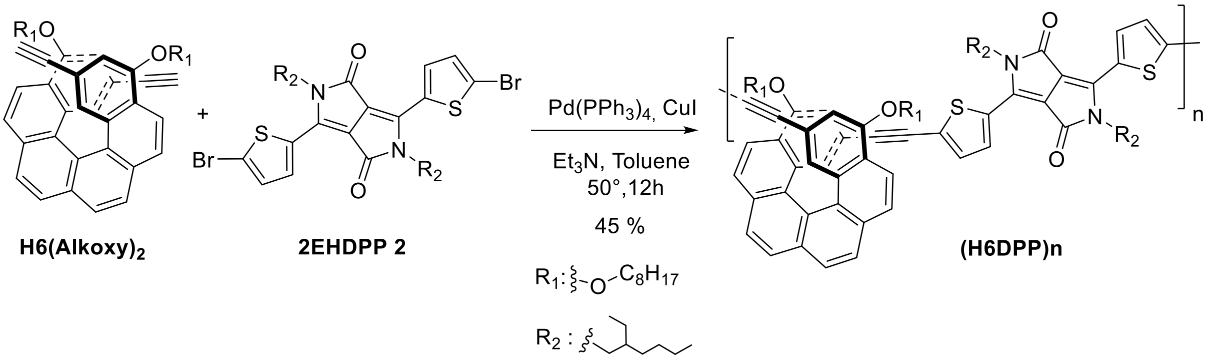


THF (6 mL) and triethylamine (2 mL) were freed from oxygen by bubbling argon for 1 hour and were then added to a mixture of compound **H6(Alkoxy)₂** (20 mg, 0.032 mmol), **2EHDPP 2** (23.80 mg ,0.035 mmol), $Pd(PPh_3)_4$ (3.60 mg, 0.0032 mmol), and CuI (1.21 mg, 0.0064 mmol) were mixed together and magnetically stirred at room temperature for 10 min and then heated at 45 ºC for 12 h. The resultant blue mixture was poured into 50 mL methanol and washed with 50 mL of acetone. The resulting blue solid was isolated by filtration and was further purified by size-exclusion chromatography (BioBeads SX-2, $CHCl_3$) to yield the final polymer (18 mg, 45 %).

**$^1$H NMR (300 MHz, Chloroform-d)** δ 9.13 – 8.89 (d, *J* = 27.2 Hz, 2H), 8.62 – 8.33 (d, *J* = 8.7 Hz, 2H), 8.24 – 7.81 (d, *J* = 10.5 Hz, 6H), 7.50 – 7.30 (d, *J* = 17.6 Hz, 4H), 6.86 – 6.65 (d, *J* = 19.1 Hz, 2H), 4.26 – 3.91 (s, 8H), 2.12 – 1.71 (d, *J* = 45.8 Hz, 8H), 1.69 – 1.08 (m, 46H), 1.09 – 0.66 (m, 20H).

# C. NMR spectra

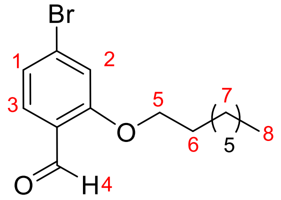


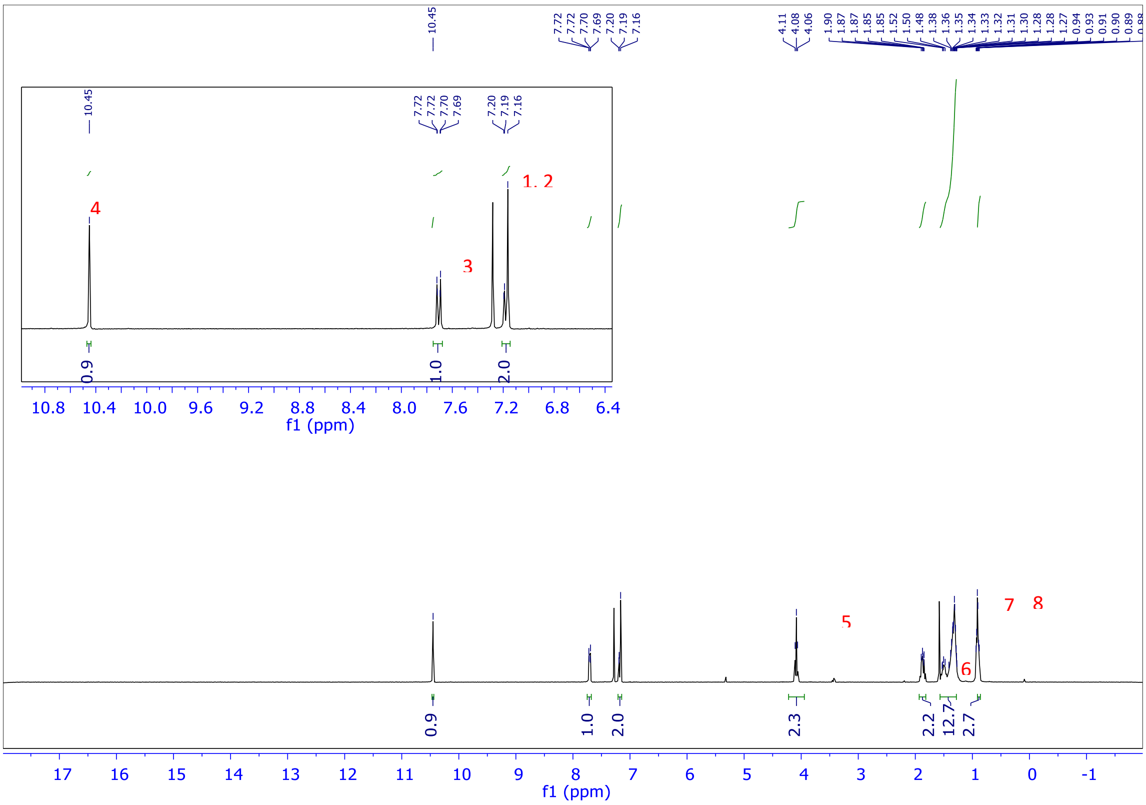


**Figure S1.** $^{1}$H NMR spectrum of (**4-bromo-2-(octyloxy)benzaldehyde)** in $CDCl_3$ at 298 K (400 MHz).

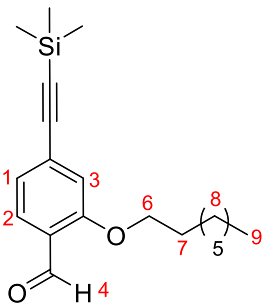

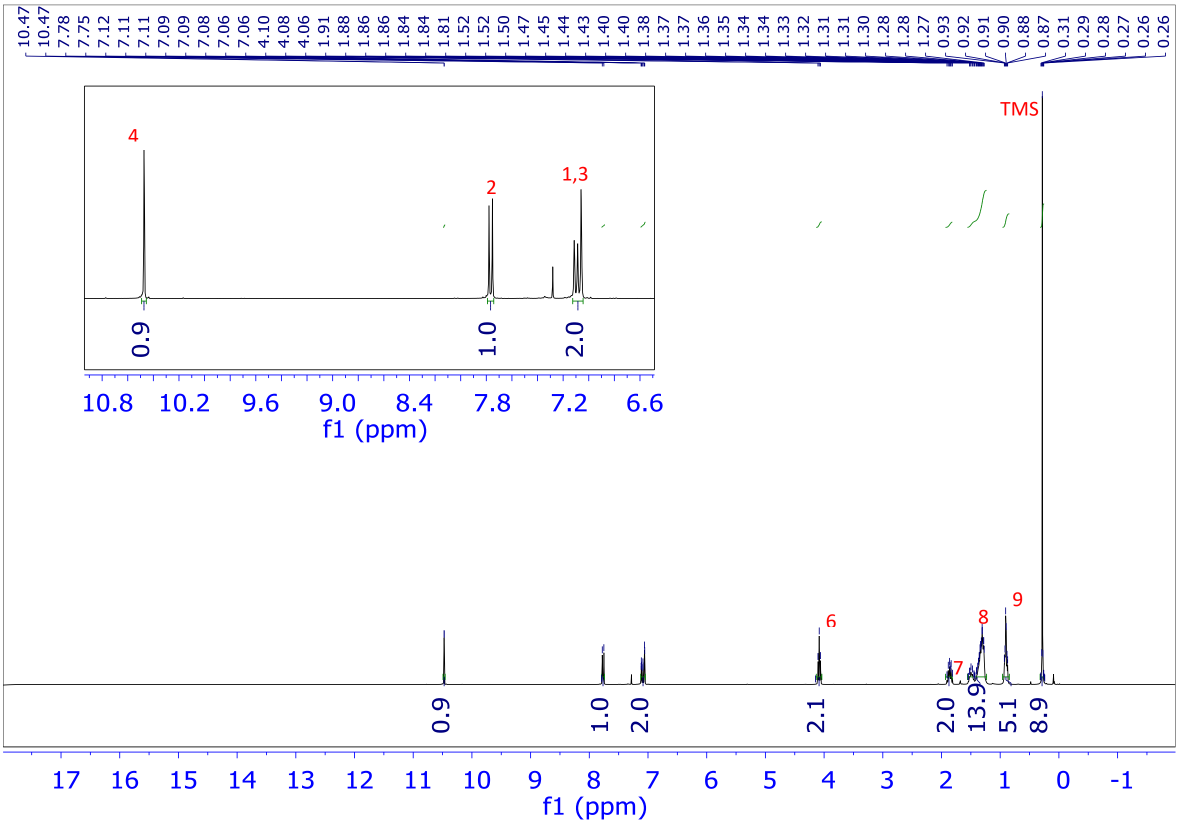


**Figure S2.** $^{1}$H NMR spectrum of **2** in $CDCl_3$ at 298 K (400 MHz).

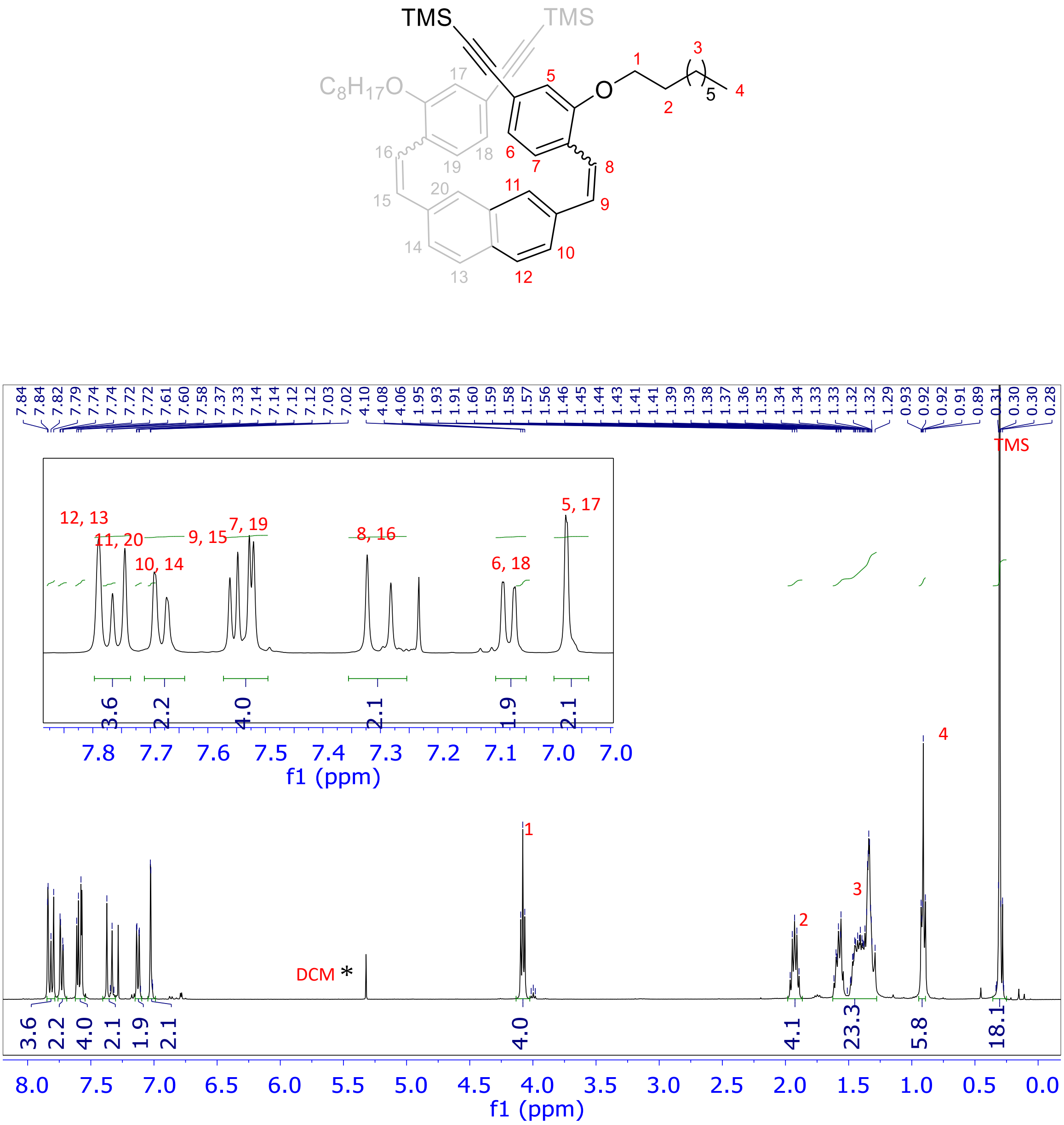

**Figure S3.** $^{1}H$ NMR spectrum of **3** in $CDCl_3$ at 298 K (400 MHz).

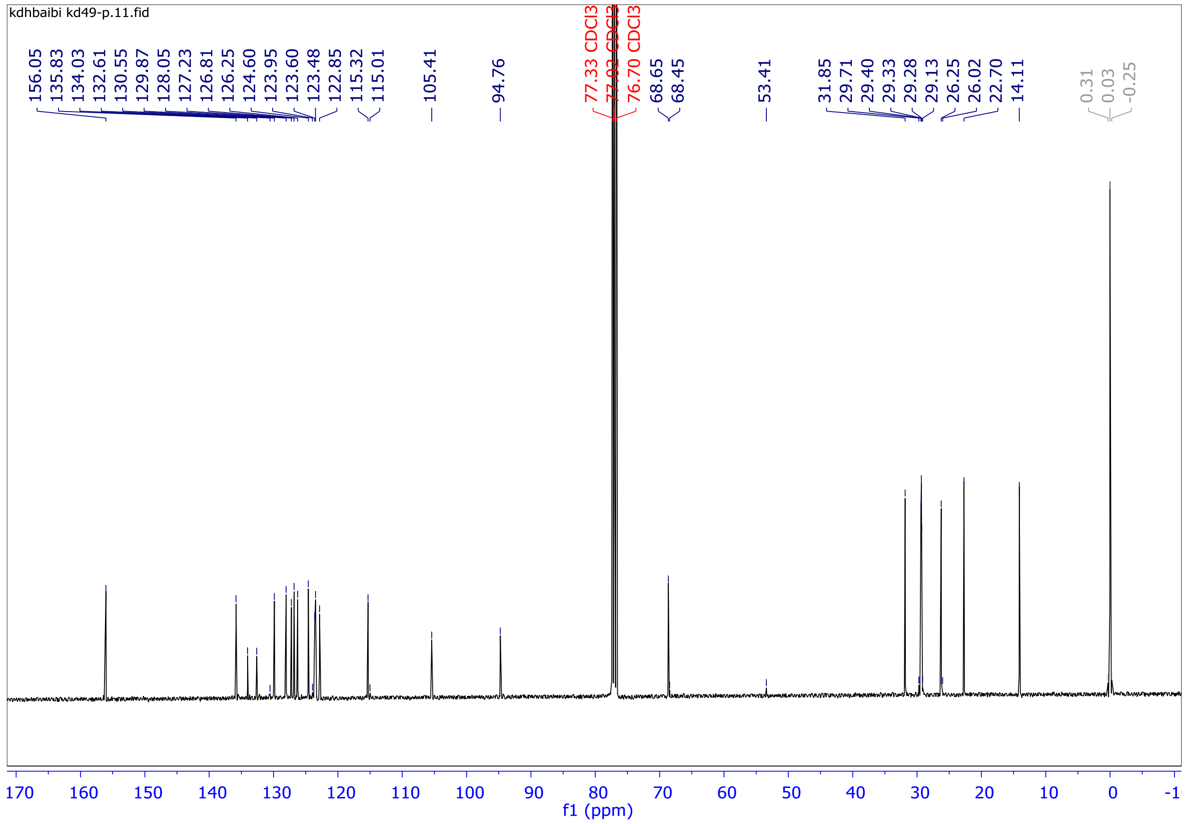


**Figure S4.** $^{13}C$ NMR spectrum of **3** in $CDCl_3$ at 298 K (101 MHz).

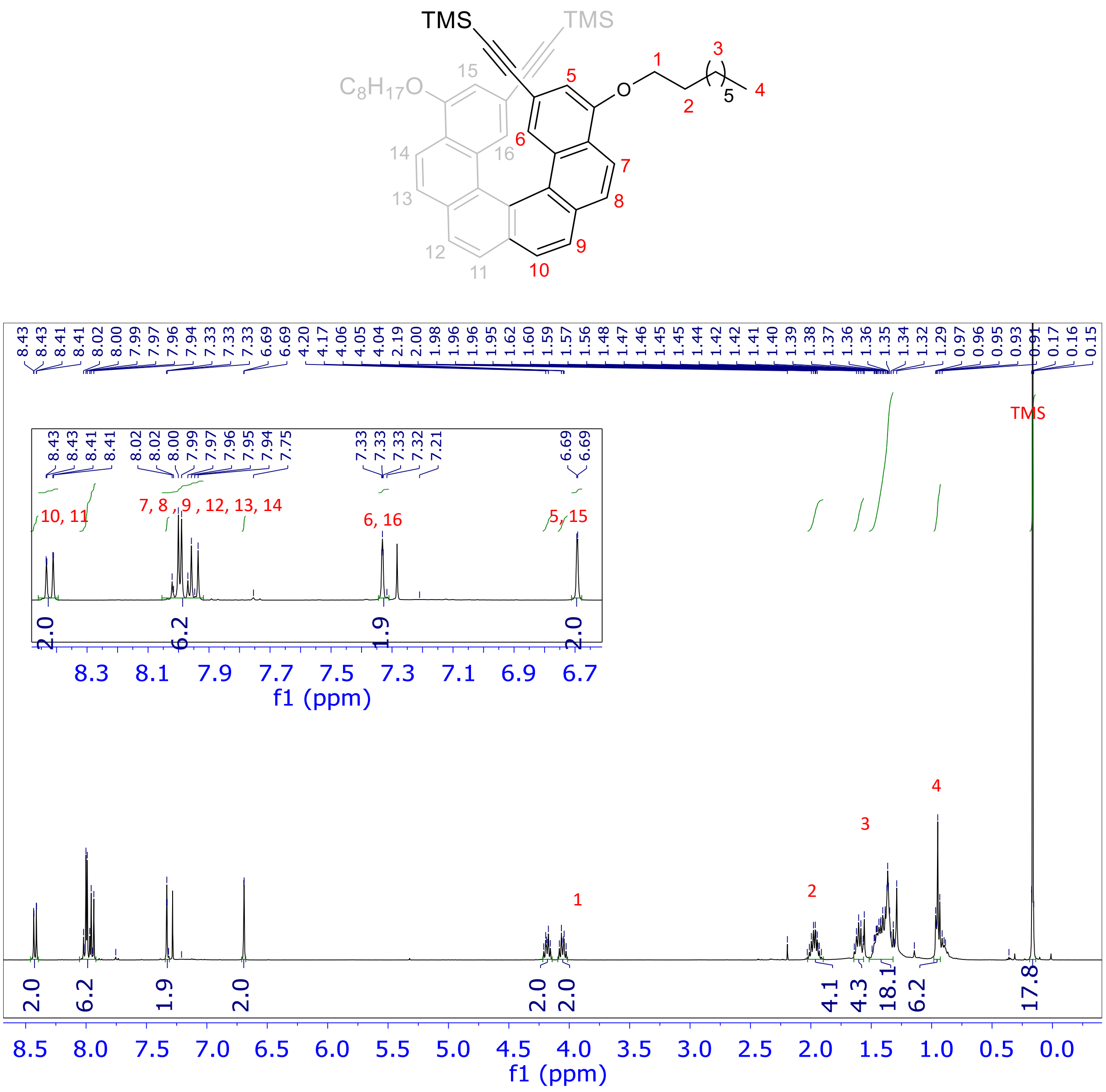

**Figure S5.** $^1$H NMR spectrum of **4** in $CDCl_3$ at 298 K (400 MHz).

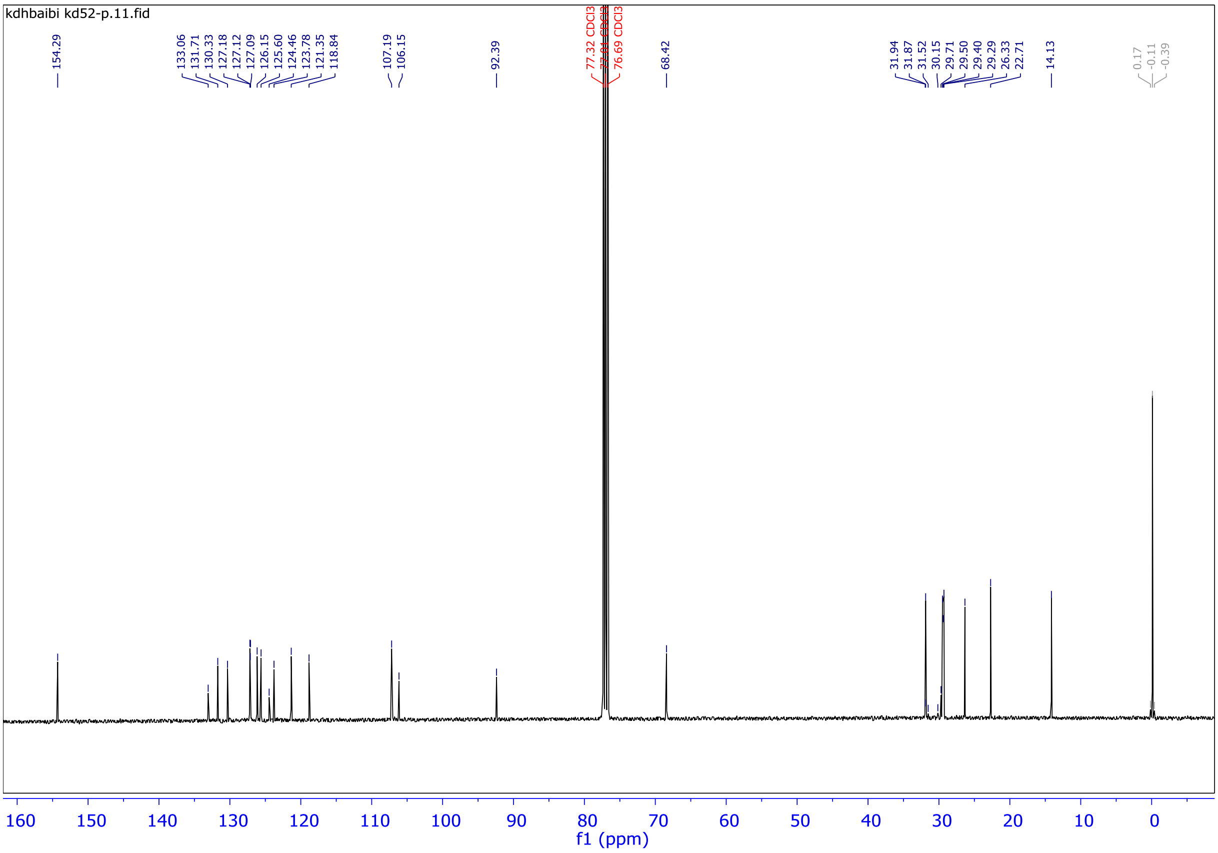


**Figure S6.** $^{13}C$ NMR spectrum of **4** in $CDCl_3$ at 298 K (101MHz).

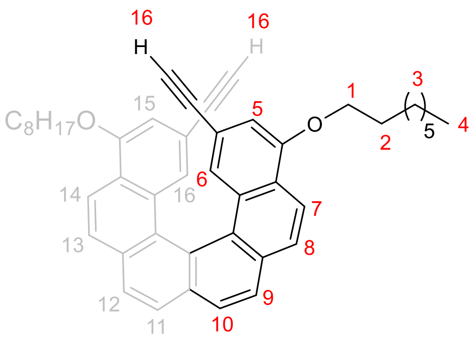

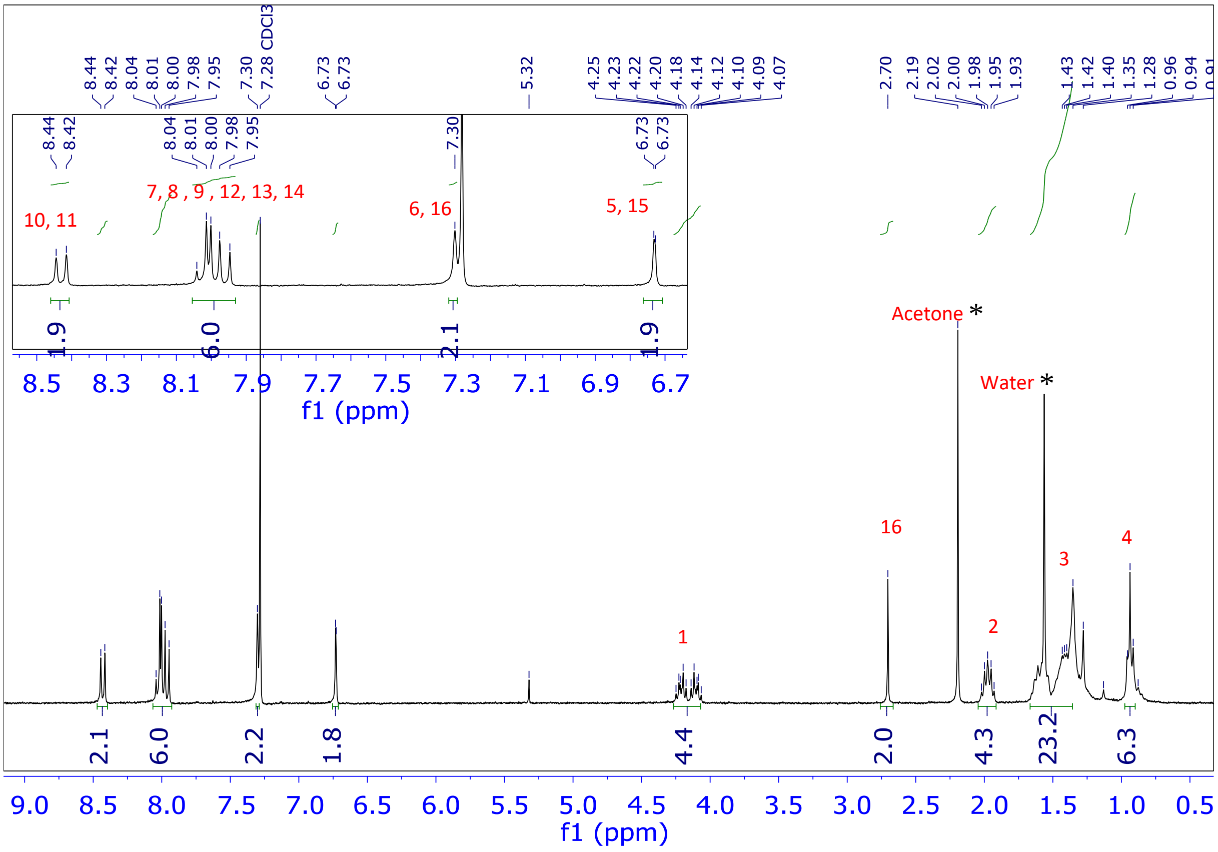


**Figure S7.** $^{1}$H NMR spectrum of **H6(Alkoxy)₂** in $CDCl_3$ at 298 K (300 MHz).

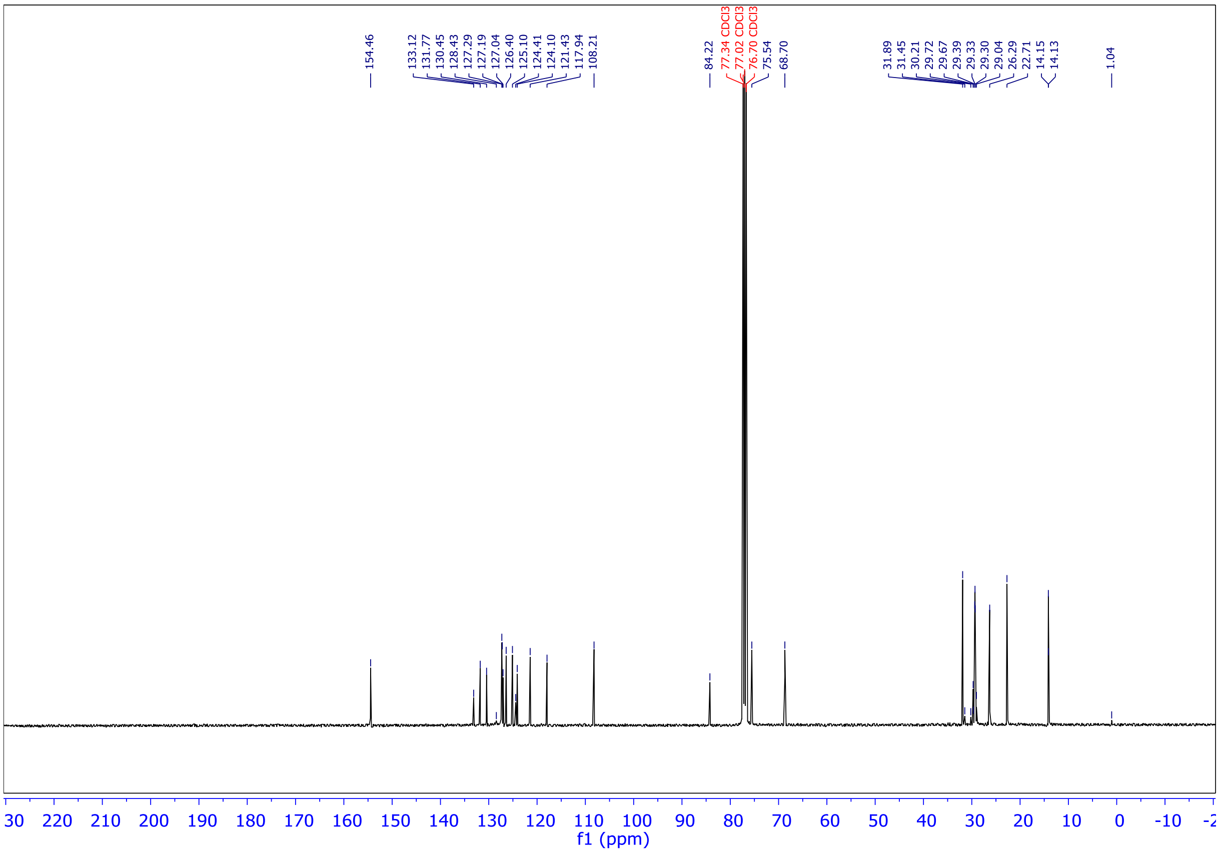


**Figure S8.** 13C NMR spectrum of **H6(Alkoxy)2** in $CDCl_3$ at 298 K (101 MHz).

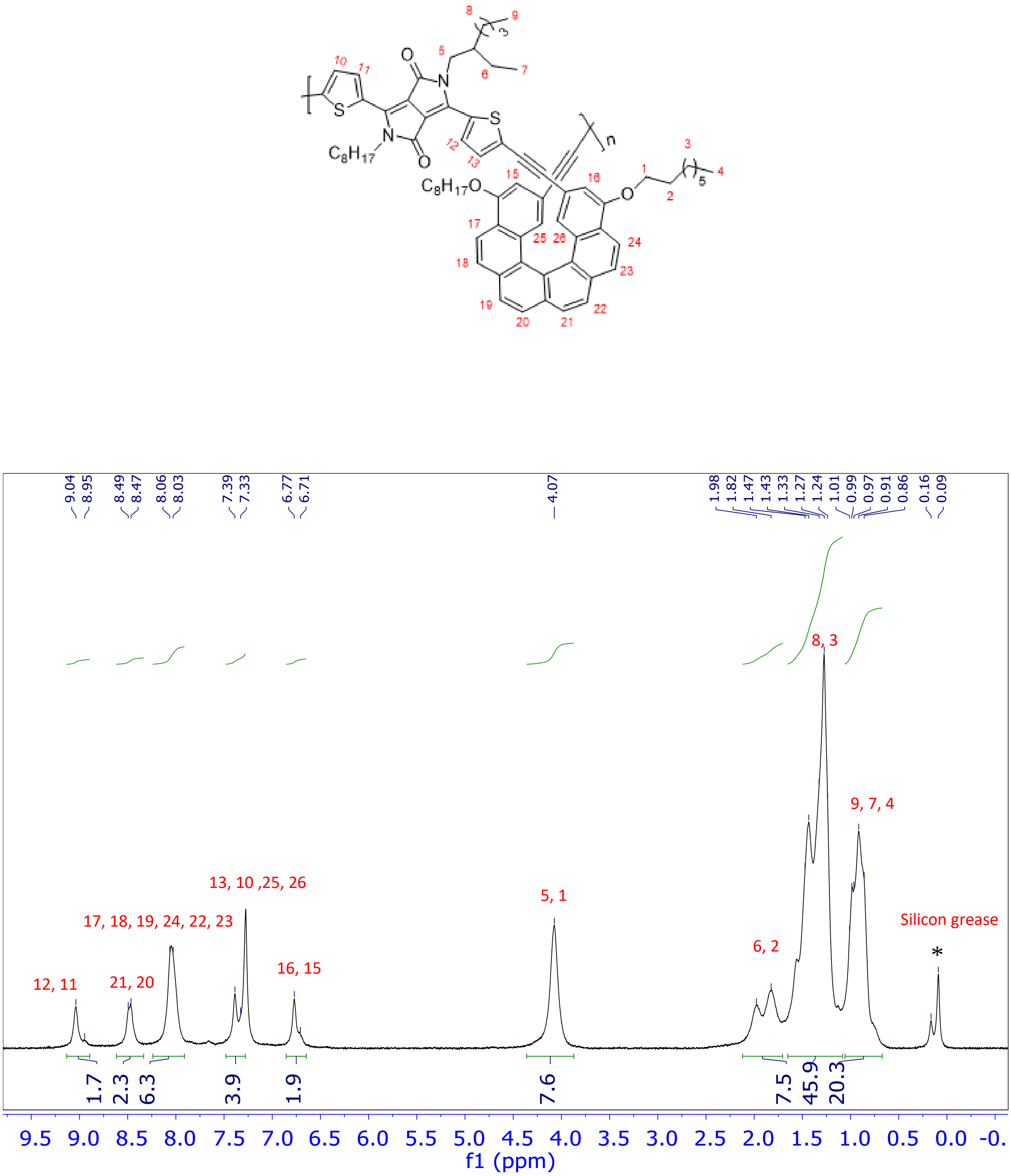


**Figure S9**. $^{1}$H NMR spectrum of polymer **$(H6DPP)_n$** in $CDCl_3$ at 298 K (300 MHz).

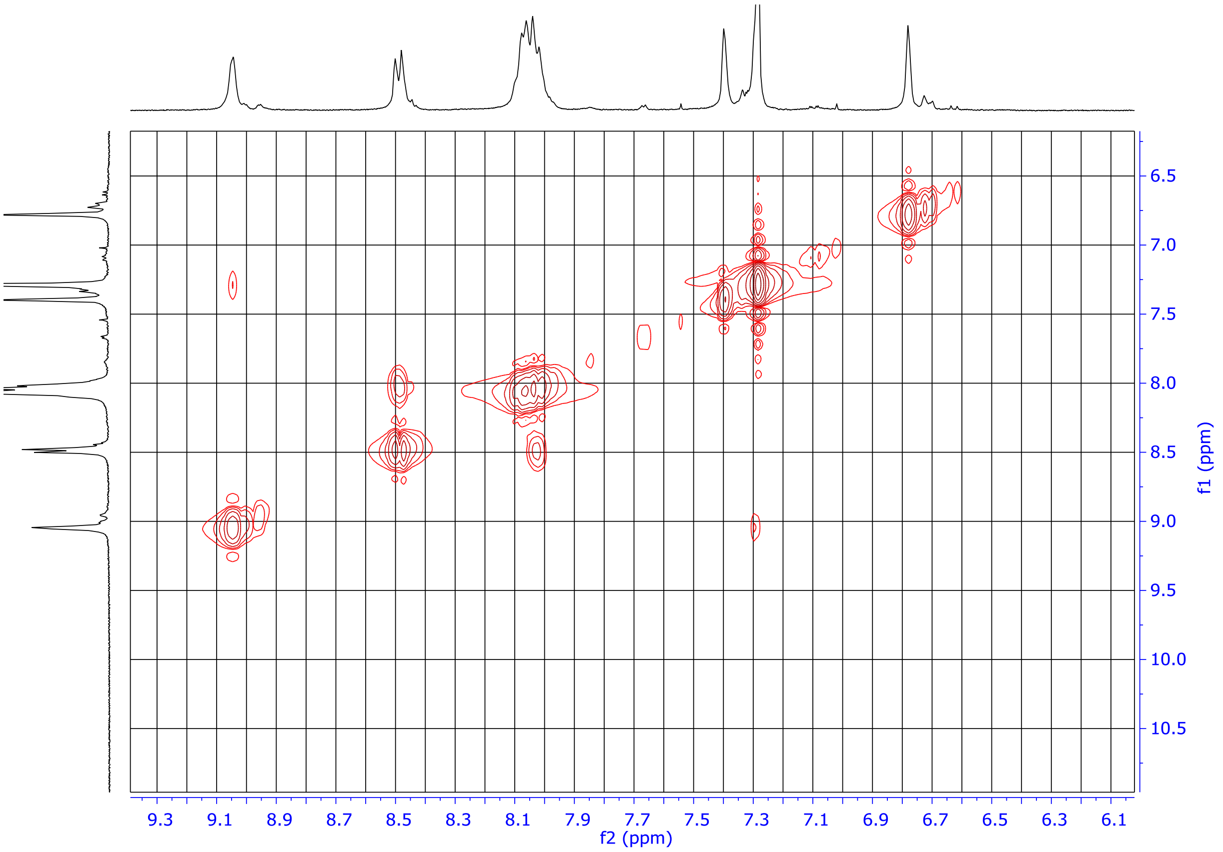


**Figure S10.** Region of the COSY spectrum of polymer **(H6DPP)$_n$** corresponding to the aromatic protons (400 MHz, $CDCl_3$, 298 K).

# D. GPC DATA

**Table S1**. Number-average and weight-average molecular weights ($M_n$ and $M_w$, respectively) and weight-average polydispersity indexes (PDI = $M_w/M_n$) of obtained polymers.

| Data | Racemic polymer | Enantiomer *P* polymer | Enantiomer *M* polymer |
|---|---|---|---|
| $M_w$ | 13065 | 8813 | 10521 |
| $M_n$ | 6916 | 5346 | 6453 |
| PDI | 1.8891 | 1.647 | 1.635 |

# E. Photophysical characterization

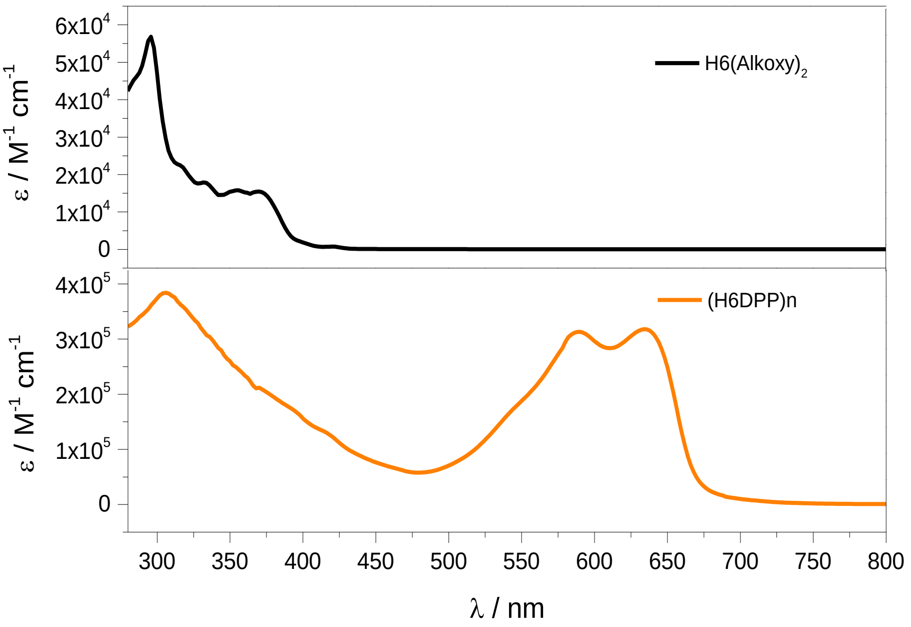


**Figure S11.** Epsilon spectra of **H6(Alkoxy)₂** (black) and **(H6DPP)ₙ** (orange) in dichloromethane solution (~$10^{-6}$ M) at 298 K.

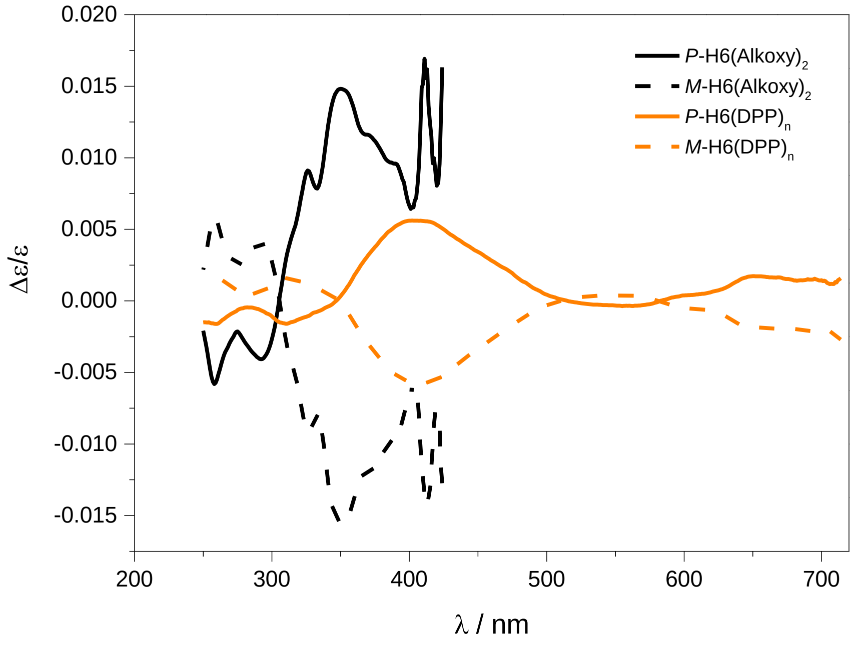


**Figure S12**. Absorption dissymmetry factor gabs spectra of compound **H6(Alkoxy)₂** (black) and the polymer **(H6DPP)ₙ** (orange) in dichloromethane solution at 298 K.

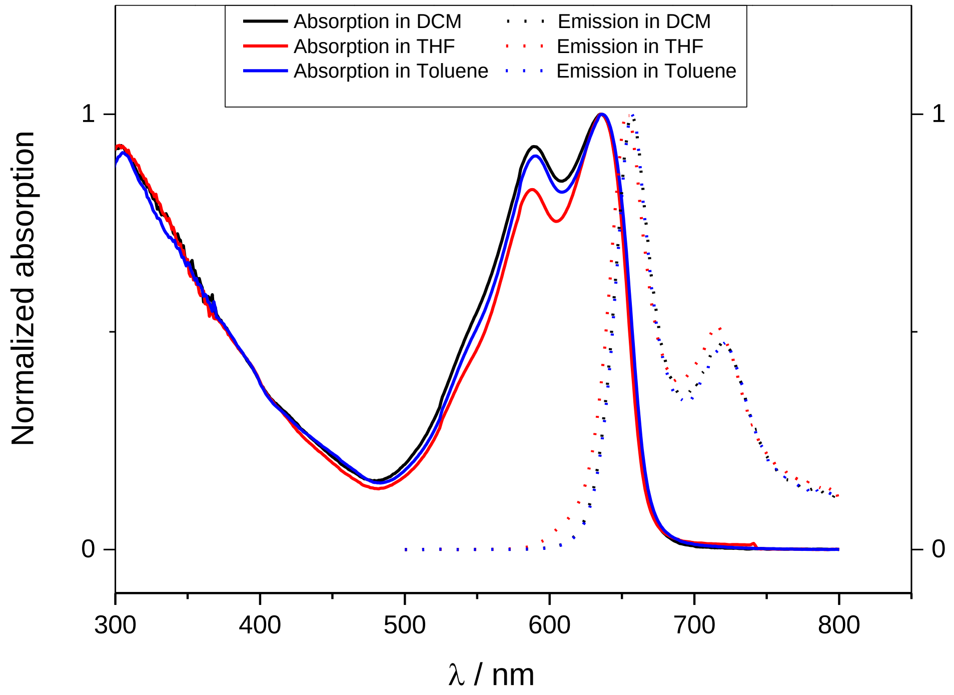


**Figure S13.** Normalized UV-vis spectra and fluorescence spectra of **H6(DPP)n** in Toluene (blue), DCM (black) and THF (red) solutions (~$10^{-6}$ M) at 298 K.

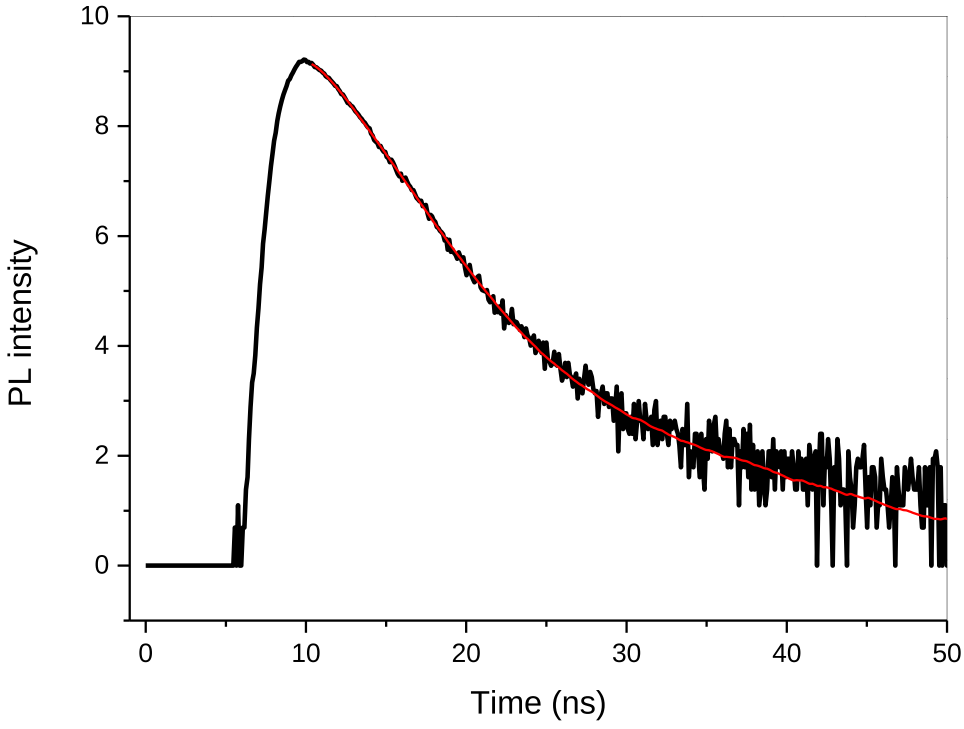


**Figure S14.** Decay profile of the fluorescence lifetimes in a DCM solution (black). The fit is also shown by the solid lines (red).

**Table S2**. Quantum yields $\Phi_f$ (averaged values) of fluorescence for the reported polymer (*rac*-, *M*-, *P*-**H6(DPP)$_n$**) and the monomer **H6(Alkoxy)$_2$**.

| Compound | $\Phi_f$ |
|---|---|
| *rac*-**H6(DPP)$_n$** | 35[a] |
| *M*-**H6(DPP)$_n$** | 35[a] |
| *P*-**H6(DPP)$_n$** | 35[a] |
| **H6(Alkoxy)$_2$** | 3[b] |

[a] relative to rhodamine 6G in ethanol

[b] relative to quinine sulphate in 0.5 M sulphuric acid

# F. Electrochemical Measurement

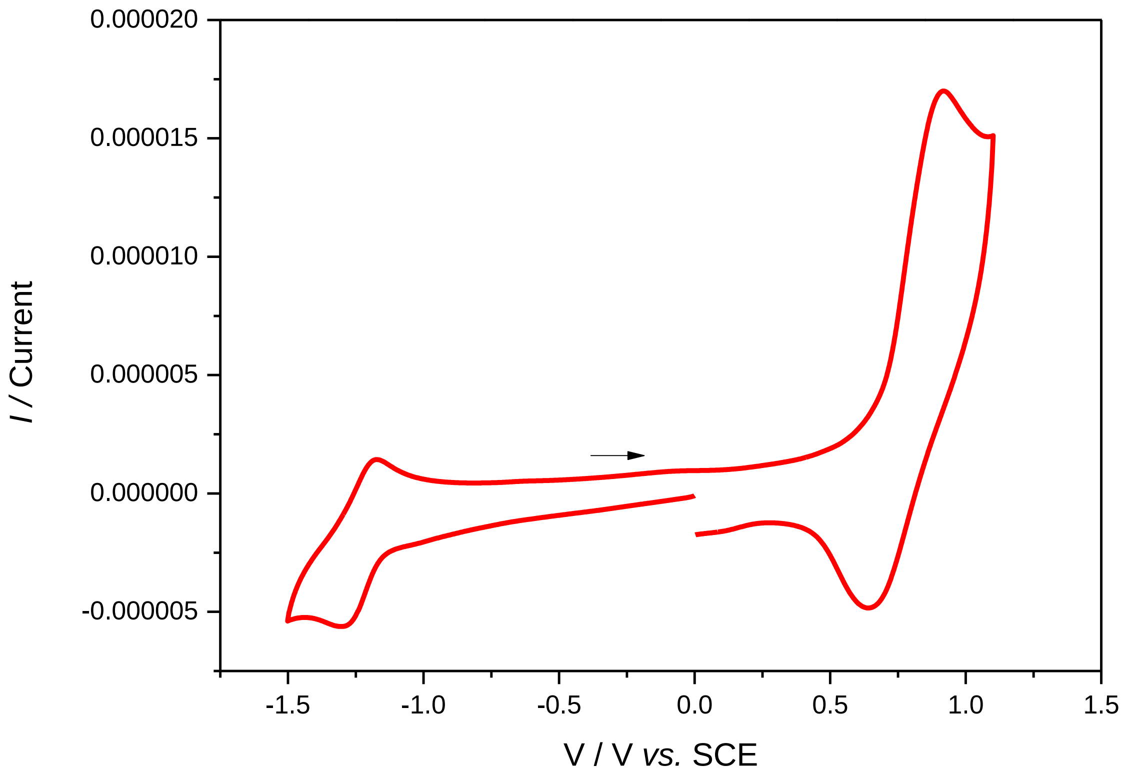


**Figure S15**. Cyclic voltammogram of **H6(DPP)$_n$** versus saturated calomel electrode (SCE) as the reference and 0.1 M $Bu_4NPF_6$ in dichloromethane as the electrolyte.

**Table S3**. Redox potentials ($E_{Ox}$ and $E_{Red}$) of **H6(DPP)$_n$** referenced versus saturated calomel electrode (SCE) and given in V.

| Compound | $E_{Ox}$ | $E_{Red}$ | HOMO[a] (eV) | LUMO[b] (eV) | $E_g$[c] (eV) |
|---|---|---|---|---|---|
| **H6(DPP)$_n$** | +0.81 | -1.24 | -5.21 | -3.16 | 2.05 |

[a] HOMO energy levels estimated from electrochemical results using the following equation: HOMO = $-(E_{Ox} + 4.4)$ eV[3]

[b] LUMO energy levels estimated from electrochemical results using the following equation: LUMO = $-(E_{Red} + 4.4)$ eV[3]

[c] Energy gaps ($E_g$) values estimated from the difference between LUMO and HOMO energy levels

# G. Thermal properties

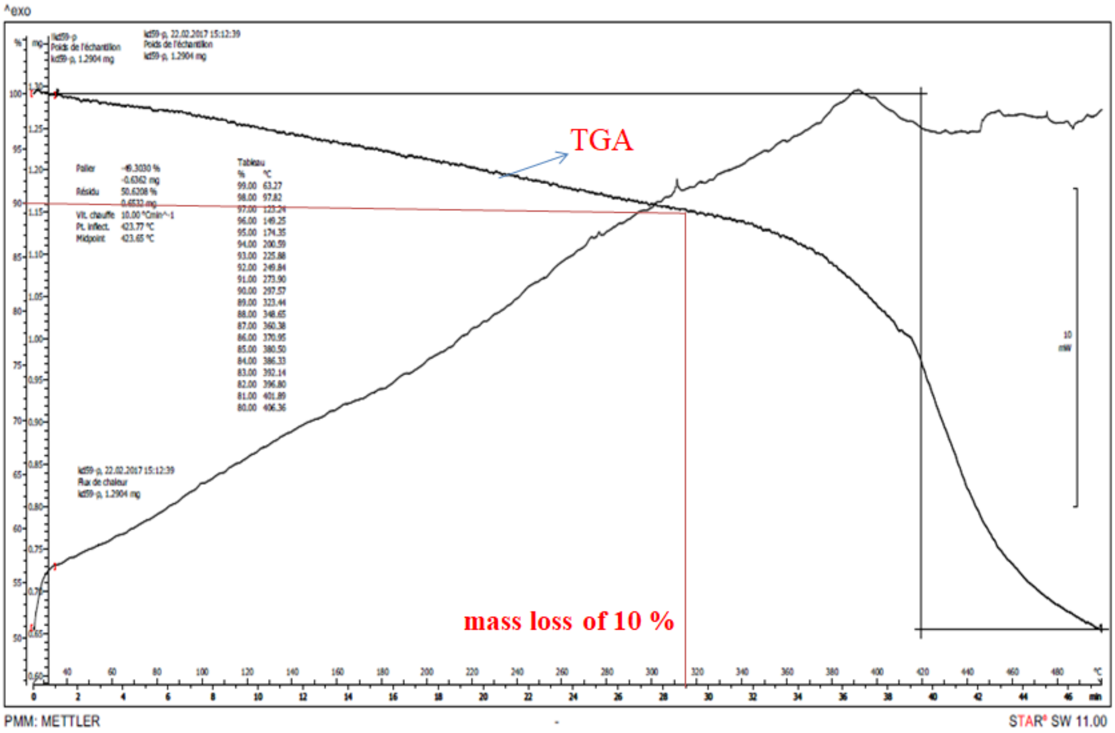


**Figure S16.** TGA plots of the polymers **H6(DPP)$_n$** with a heating rate of 10 °C/min under argon.

# H. HPLC separations

*- Analytical chiral HPLC separation for* **H6(Alkoxy)$_2$**

• The sample is dissolved in dichloromethane, injected on the chiral column, and detected with an UV detector at 254 nm and CD 300 nm. The flowrate is 1 mL/min.

| Column | Mobile Phase | t1 | k1 | t2 | k2 | α | Rs |
|---|---|---|---|---|---|---|---|
| **Chiralpak IF** | Heptane / 2-PrOH (98/2) | 4.52 (*P*) | 0.53 | 5.06 (*M*) | 0.72 | 1.32 | 2.20 |

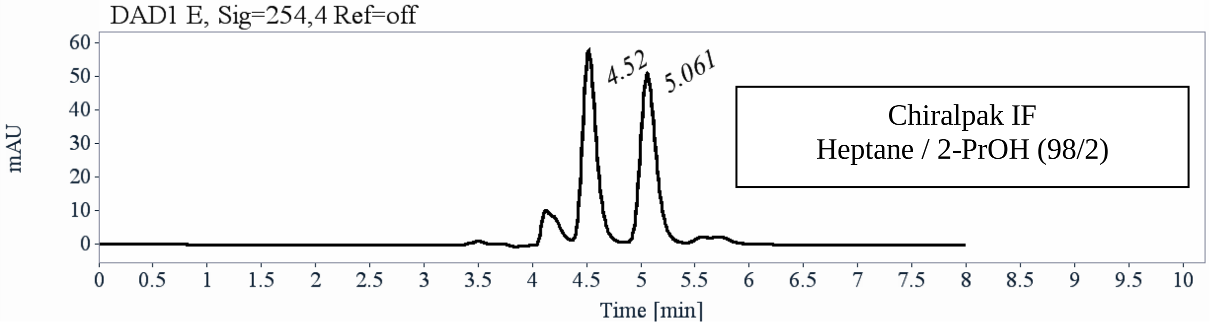


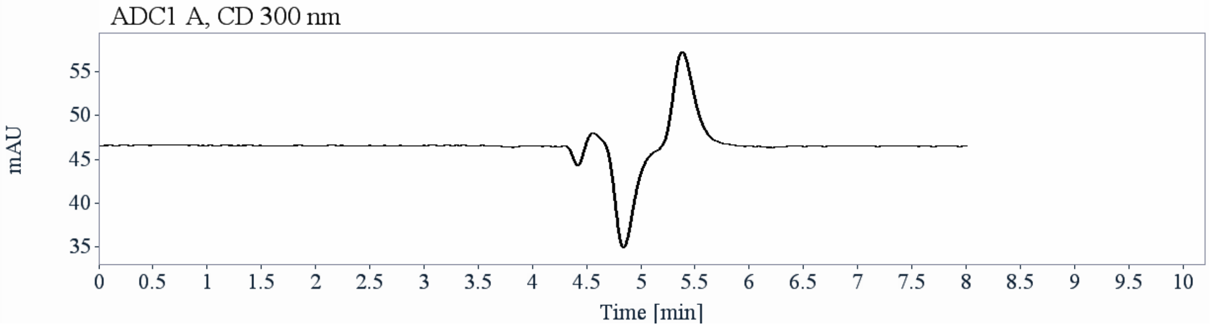


| RT [min] | Area | Area% | Capacity Factor | Enantioselectivity | Resolution (USP) |
|---|---|---|---|---|---|
| 4.52 | 501 | 50.03 | 0.53 | | |
| 5.06 | 500 | 49.97 | 0.72 | 1.34 | 2.20 |
| Sum | 1001 | 100.00 | | | |

***Preparative separation for compound*** **H6(Alkoxy)$_2$:**

• Sample preparation: About 300 mg of compound **H6(Alkoxy)$_2$** are dissolved in 15 mL of dichloromethane.

• Chromatographic conditions: Chiralpak IF (250 x 10 mm), hexane / 2-PrOH (95/5) as mobile phase, flowrate = 5 mL/min, UV detection at 254 nm.

• Injections: 750 times 20 µL, every 1.5 minutes.

• Collection: the first eluted enantiomer is collected between 3.9 and 4.2 minutes, the second one between 4.4 and 4.8 minutes.

• First fraction: 125 mg of the first eluted (*P*)-enantiomer with ee > 99%

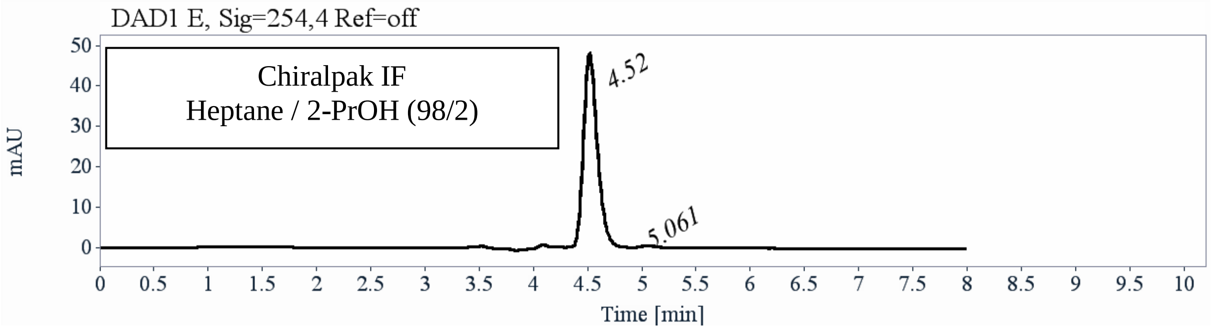


| RT [min] | Area | Area% |
|---|---|---|
| 4.52 | 456 | 99.56 |
| 5.06 | 2 | 0.44 |
| Sum | 458 | 100.00 |

• Second fraction: 100 mg of the second eluted (*M*)-enantiomer with ee > 99%

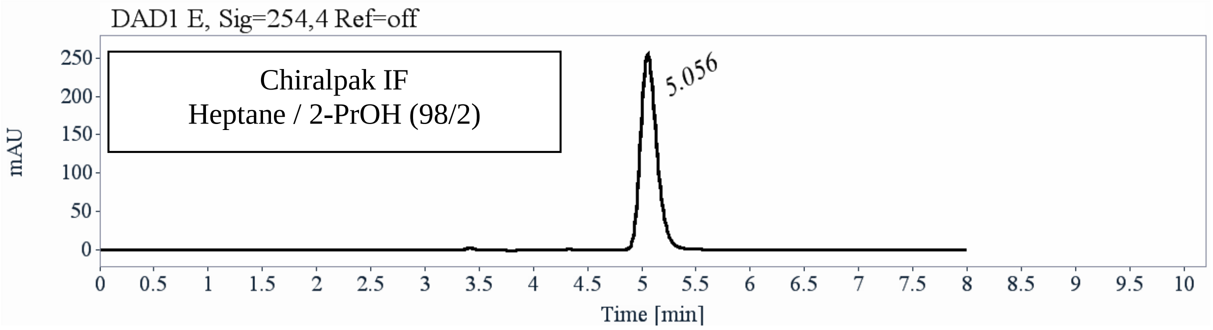


| RT [min] | Area | Area% |
|---|---|---|
| 5.06 | 2654 | 100.00 |
| Sum | 2654 | 100.00 |

# I. X-ray crystallographic data

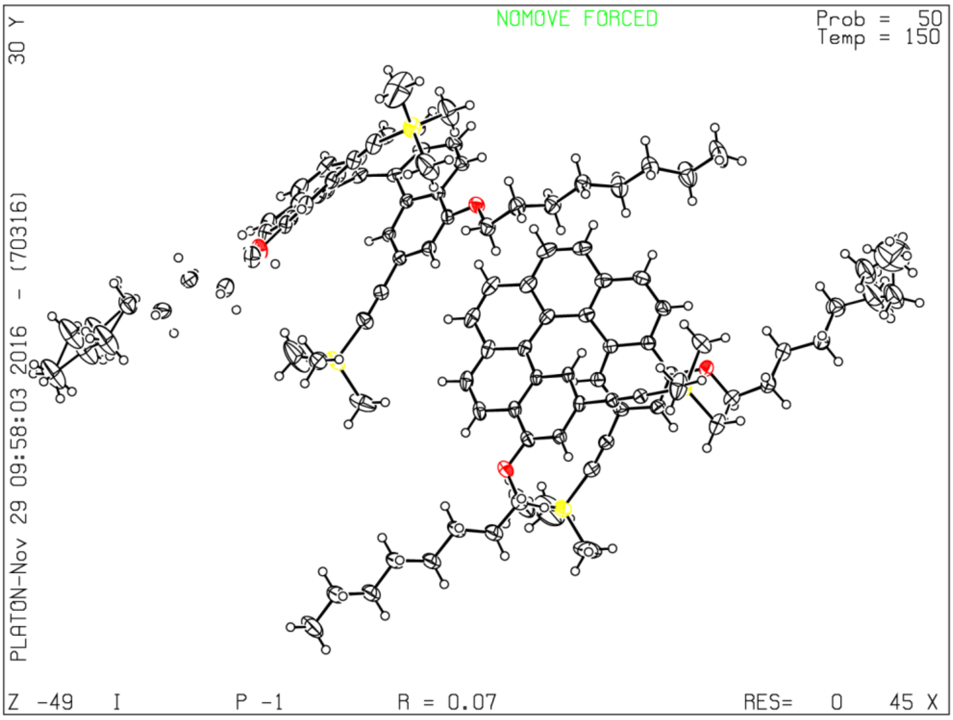


**Figure S18**. ORTEP diagrams of compound **4** with ellipsoids at 50% probability (at 100 K).

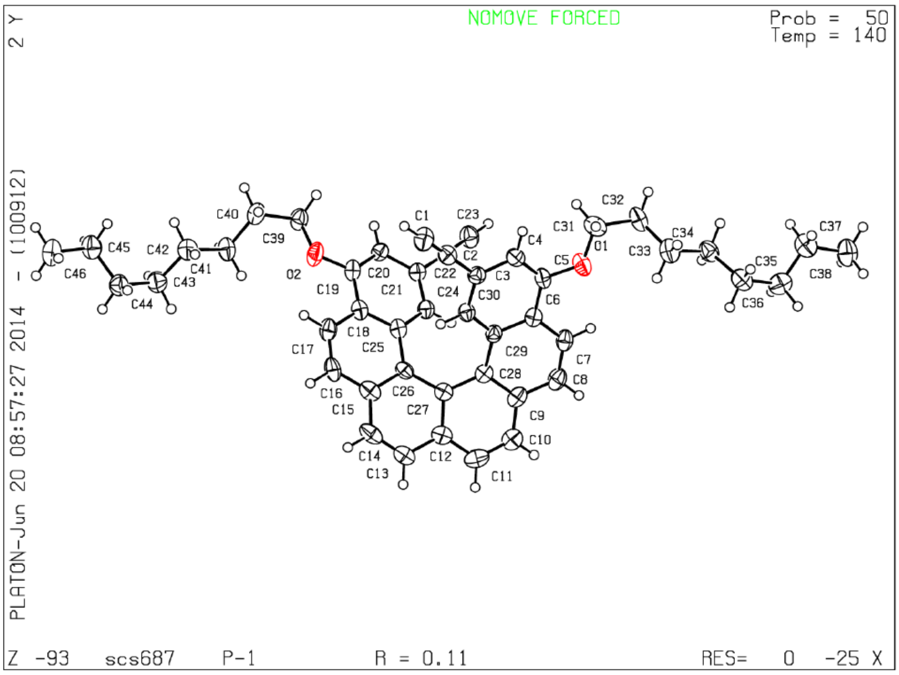


**Figure S19**. ORTEP diagrams of compound **H6(Alkoxy)$_2$** with ellipsoids at 50% probability (at 100 K).

**Table S4**. Crystallographic data for **4** and **H6(Alkoxy)$_2$**

| | **4** | **H6(Alkoxy)$_2$** |
|---|---|---|
| Empirical formula | C52H64O2Si2 | C46H48O2 |
| CCDC number | 1951648 | 1009994 |
| Formula weight (g/mol) | 777.21 | 632.84 |
| Temperature (K) | 150 | 140 |
| Wavelength (Å) | 0.71073 | 0.71073 |
| Crystal system | Triclinic | Triclinic |
| Space group | *P*-1 | *P*-1 |
| *a* (Å) | 13.9607(7) | 9.1951(6) |
| *b* (Å) | 15.9349(7) | 9.868(2) |
| *c* (Å) | 22.1109(9) | 21.711(3) |
| α(°) | 92.330(3) | 93.590(10) |
| β(°) | 105.843(2) | 91.640(10) |
| γ(°) | 93.793(2) | 115.330(10) |
| Volume (Å$^3$) | 4712.8(4) | 1773.7(5) |
| *Z* | 4 | 2 |
| $\rho_{calculated}$ (g.cm$^{-3}$) | 1.095 | 1.185 |
| Absorption coefficient (mm$^{-1}$) | 0.112 | 0.070 |
| *F(000)* | 1680 | 680.0 |
| Crystal size (mm) | 0.450 x 0.320 x 0.060 | 0.268 x 0.103 x 0.101 |
| Crystal color | Colourless | colourless |
| $\theta$ range for data collection (°) | 2.927 to 27.458 | 3.08 to 27.00 |
| Limiting indices | -18≤h≤18 | -11≤h≤11 |
| | -20≤k≤20 | -11≤k≤12 |
| | -28≤l≤28 | -27≤l≤27 |
| Reflections unique | 69827 / 21240 [R(int) = 0.0875] | 14556 / 7730 [R(int) = 0.1105] |
| Reflections collected [I>2s(I)] | 13362 | 2664 |
| Completeness to $\theta$_max | 0.985 | 0.998 |
| Absorption correction type | multi-scan | multi-scan |
| Max. and min. transmission | 0.993 , 0.783 | 0.993 , 0.783 |
| Refinement method | Full-matrix least-squares on F^2 | Full-matrix least-squares on F^2 |
| Data / restraints / parameters | 21240 / 0 / 1020 | 7730 / 0 / 433 |
| Goodness-of-fit on *F*2 | 1.034 | 1.010 |
| Final R indices [*I*>2s (*I*)] | R1 = 0.0712, wR2 = 0.1696 | R1 = 0.1130, wR2 = 0.2511 |
| R indices (all data) | R1 = 0.1248, wR2 = 0.2027 | R1 = 0.2759, wR2 = 0.3592 |
| Largest diff. peak and hole (e Å$^{-3}$) | 0.891 and -1.042 | 0.501 and -0.354 |

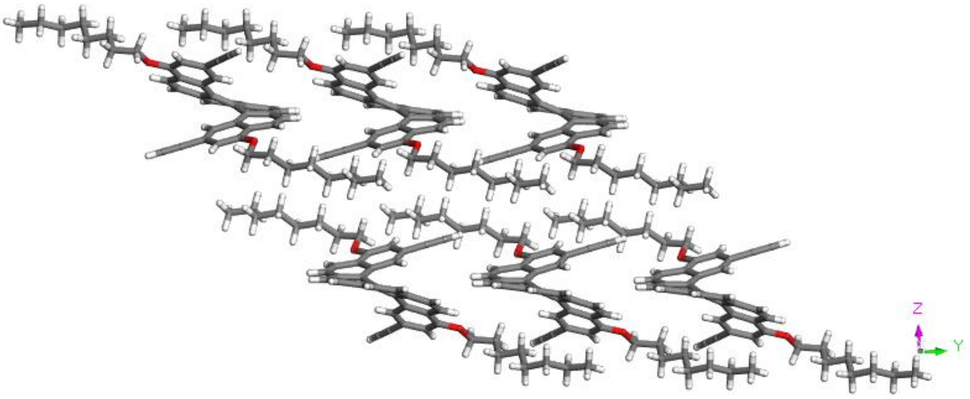


**Figure S20.** Arrangement of **H6(Alkoxy)$_2$** in the solid state.

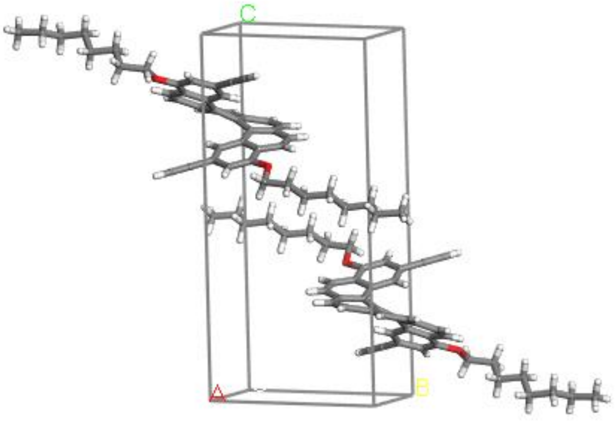


**Figure S21.** Heterochiral arrangement of **4** in the solid state (unit cell).

# J. References

[1] C. Würth, M. Grabolle, J. Pauli, M. Spieles and U. Resch-Genger, *Nat. Protoc.* **2013**, *8*, 1535.
[2] A. K. Palai, S. P. Mishra, A. Kumar, R. Srivastava, M. N. Kamalasanan and M. Patri, *Macromol. Chem. Phys.* **2010**, *211*, 1043-1053.
[3] C. M. Cardona, W. Li, A. E. Kaifer, D. Stockdale and G. C. Bazan, *Adv. Mater.* **2011**, *23*, 2367-2371.
